# A systematic review reveals conflicting evidence for the prevalence of antibodies against the sialic acid ‘xenoautoantigen’ Neu5Gc in humans and the need for a standardised approach to quantification

**DOI:** 10.3389/fmolb.2024.1390711

**Published:** 2024-04-26

**Authors:** Esme Hutton, Emma Scott, Craig N. Robson, Nathalie Signoret, Martin A. Fascione

**Affiliations:** ^1^ Department of Chemistry, University of York, York, United Kingdom; ^2^ Hull York Medical School, University of York, York, United Kingdom; ^3^ Newcastle University, Centre for Cancer, Newcastle University Biosciences Institute, Newcastle, United Kingdom; ^4^ Newcastle University, Centre for Cancer, Newcastle University Translational and Clinical Research Institute, Newcastle, United Kingdom

**Keywords:** Neu5Gc, anti-Neu5Gc antibodies, xeno-autoantigen, sialic acid, glycoscience

## Abstract

Despite an array of hypothesised implications for health, disease, and therapeutic development, antibodies against the non-human sialic acid *N*-glycolylneuraminic acid (Neu5Gc) remain a subject of much debate. This systematic review of 114 publications aimed to generate a comprehensive overview of published studies in this field, addressing both the reported prevalence of anti-Neu5Gc antibodies in the human population and whether experimental variation accounts for the conflicting reports about the extent of this response. Absolute titres of anti-Neu5Gc antibodies, the reported prevalence of these antibodies, and the individual variation observed within experiments were analysed and grouped according to biological context (‘inflammation’, ‘xenotransplantation’, ‘biotherapeutic use’, ‘cancer’, and ‘healthy populations’), detection method, target epitope selection, and choice of blocking agent. These analyses revealed that the experimental method had a notable impact on both the reported prevalence and absolute titres of anti-Neu5Gc antibodies in the general population, thereby limiting the ability to ascribe reported trends to genuine biological differences or the consequence of experimental design. Overall, this review highlights important knowledge gaps in the study of antibodies against this important xenoautoantigen and the need to establish a standardised method for their quantification if the extent of the importance of Neu5Gc in human health is to be fully understood.

## Introduction

### The sialic acid landscape in humans

**FIGURE 1:**
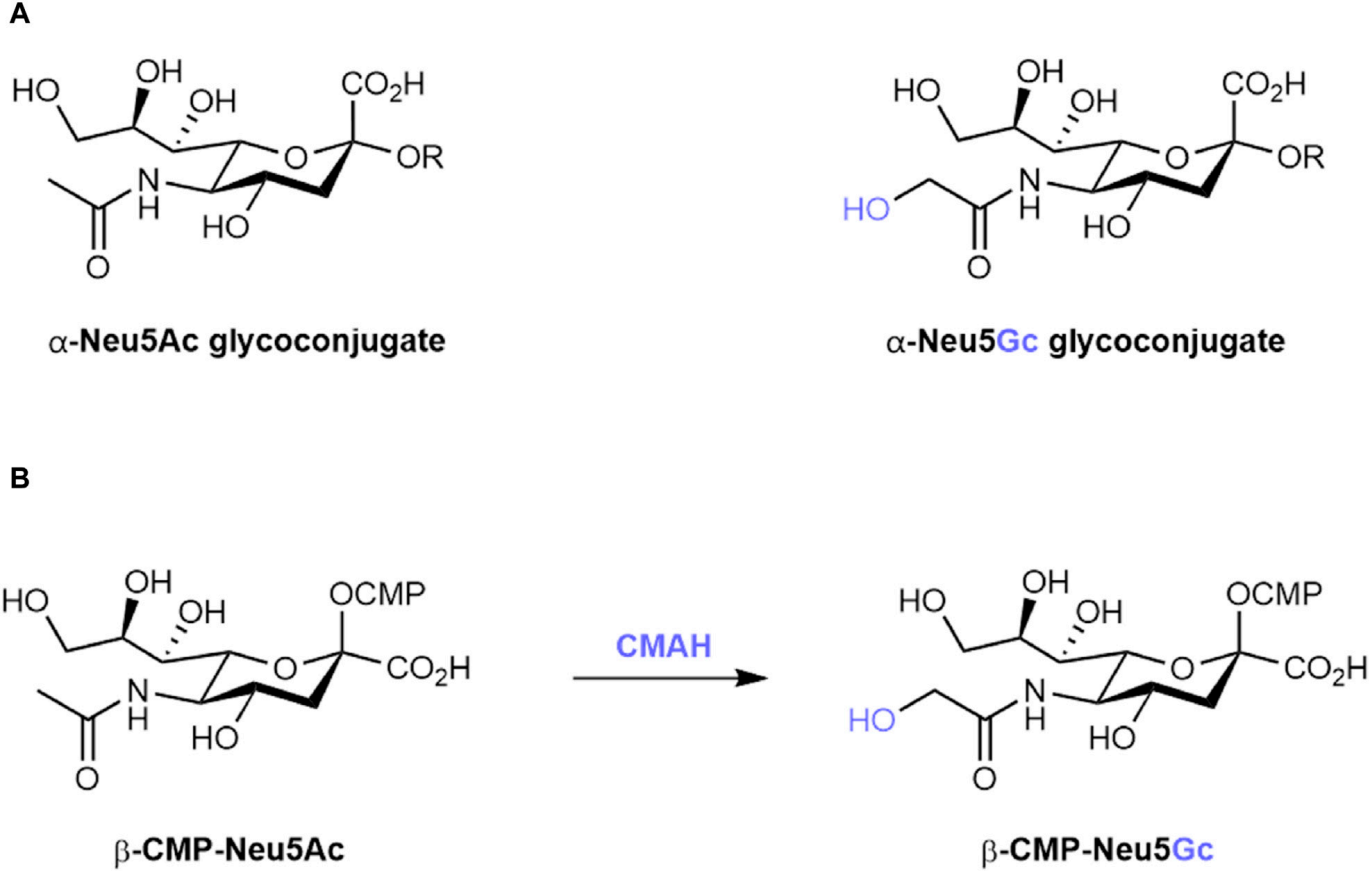
**(A)** Structures of α-linked N-acetylneuraminic acid (Neu5Ac) and α-linked N-glycolylneuraminic acid (Neu5Gc) glycoconjugates. R represents the underlying glycan scaffold. **(B)** Biosynthetic conversion of cytidine monophosphate-activated Neu5Ac (β-CMP-Neu5Ac) to β-CMP-Neu5Gc by the CMAH enzyme.

Sialic acids are nine-carbon, negatively charged sugars often found as the terminating unit of glycan chains at the surface of many eukaryotic cells (Varki et al., 2015). Due to their position at the outermost limits of the glycocalyx, sialic acids are important in the regulation of cell–cell interactions, in shielding underlying structures from enzymatic activity, and as a frequent host receptor for a range of bacterial and viral pathogens (Varki, 2008). These sialic acids are found in two main forms across most members of the deuterostome lineage: *N*-acetylneuraminic acid (Neu5Ac) and, differing by a single oxygen atom, *N*-glycolylneuraminic acid (Neu5Gc) (Figure 1A) (Varki, 2001a).

However, in multiple species, including humans, loss-of-function mutations in cytidine monophospho-*N*-acetylneuraminic acid hydroxylase (CMAH), the enzyme responsible for the conversion of CMP-activated Neu5Ac to CMP-Neu5Gc (Figure 1B), have led to an inability to endogenously synthesise Neu5Gc (Malykh et al., 2001; Altman and Gagneux, 2019). In humans, this inactivation event is estimated to have occurred 2–3 million years ago, following the divergence of humans from our closest living ancestor, the chimpanzee (Peri et al., 2018). In two independent reports published in 1998, CMAH inactivation was traced back to a 92 base-pair deletion in exon nine of the *Cmah* gene (Chou et al., 1998; Irie and Suzuki, 1998). While the proposed consequence of this event – either a premature stop codon or a frameshift mutation – differed between the two publications, both resulted in the loss of a hypothesised Rieske iron-sulphur binding domain important for CMAH catalytic activity (Hayakawa et al., 2001). Consolidation of CMAH loss in the human population is hypothesised to be the result of selective pressure from Neu5Gc-binding pathogens such as *Plasmodium reichenowi* (Okerblom and Varki, 2017) and sexual selection leading to immune clearance of Neu5Gc-positive sperm in *Cmah* ^−/−^ female individuals (Ghaderi et al., 2011).

Despite the loss of CMAH activity and no evidence for an alternative biosynthetic pathway for Neu5Gc (Wang et al., 2023), glycan structures terminating in Neu5Gc have been reported in a range of human tissues (Tangvoranuntakul et al., 2003). Although immunologically distinct, Neu5Gc and Neu5Ac are both processed by the same enzymatic ‘sialylation machinery’, with sialyltransferases and sialic acid transporters utilising both CMP-sugars interchangeably (Varki, 2001b). This is supported by *in vitro* assays in cells treated with exogenous Neu5Gc that revealed extensive uptake and incorporation of Neu5Gc into cell surface glycans and biochemical evidence that mammalian CMP-sialic acid synthetase is also active on both sialic acids (Kean and Roseman, 1966; Tangvoranuntakul et al., 2003). Processing of Neu5Gc was also prevented in the presence of an excess of Neu5Ac (Bardor et al., 2005). Additionally, studies in *Cmah* ^−/−^ mice unable to synthesise Neu5Gc showed a reduction in Neu5Gc incorporation into aortic endothelial cells when fed competing doses of Neu5Ac and Neu5Gc (Kawanishi et al., 2021). This aligns well with studies in colorectal cancer cells highlighting that hypoxia-driven upregulation of sialic acid transport facilitates increased Neu5Gc presentation, implying that the overall rate of sialic acid turnover is a key determining factor in the amount of Neu5Gc displayed at the cell surface (Yin et al., 2006). Overall, this information has converged into a generally accepted theory that Neu5Gc found in human tissues is derived from exogenous sources, such as red meat and dairy in the diet (Dhar et al., 2019). The ability of exogenous Neu5Gc to act as a *de facto* metabolic ‘Trojan Horse’ frames it as a unique example of a xenoautoantigen: a non-human epitope that is presented on glycoconjugates at the surface of human cells (Altman and Gagneux, 2019).

### Neu5Gc as a xenoautoantigen

Neu5Gc-containing glycans are thought to be immunogenic in humans, triggering the development of a polyclonal anti-Neu5Gc humoral immune response that accommodates Neu5Gc in the context of an array of underlying glycan structures (Breimer and Holgersson, 2019). This presentation of Neu5Gc on a diverse range of underlying scaffolds differs from other carbohydrate xenoantibodies, such as anti-αGal antibodies, which are directed against a single Galα1-3Galβ1-4GlcNAc-R epitope and are triggered by early-life exposure to these glycans on commensal bacteria (Padler-Karavani and Varki, 2011; Galili, 2023). This relative simplicity may explain why αGal antibodies are reliably reported across the entire human population, whereas the extent of the anti-Neu5Gc antibody response remains controversial (Galili, 2023).

Induction of an anti-Neu5Gc antibody response in humans is thought to occur at the age of 6–12 months, typically associated with the introduction of solid foods or milk-based formula into the diet (Taylor et al., 2010). Interestingly, the induction of anti-Neu5Gc antibodies also strongly coincided with colonisation of the gut by microorganisms (Taylor et al., 2010). While bacteria do not possess the enzymatic machinery required to endogenously synthesise Neu5Gc, genetic screening of a range of both commensal and terrestrial bacteria identified an array of hypothetical sialidases capable of preferentially metabolising Neu5Gc (Zaramela et al., 2019). This aligns with *in vivo* evidence that Neu5Gc ‘scavenged’ by non-typeable *Haemophilus influenzae* (NTHi) – a common commensal microorganism – could induce an anti-Neu5Gc immune response in *Cmah* ^−/−^ mice (Taylor et al., 2010). The potential for presentation of Neu5Gc-containing epitopes on commensal or pathogenic bacteria may explain the induction of anti-Neu5Gc antibodies even in populations with a low consumption of Neu5Gc-containing animal products such as red meat and dairy (Zaramela et al., 2019).

### Anti-Neu5Gc antibodies in human disease

Because of its unique position as a xenoautoantigen, the potential implications for Neu5Gc in human health have been an area of interest for almost a century. Hanganutziu–Deicher (HD) antibodies were described in the 1920s as a driver of serum sickness in diphtheria patients treated with animal antisera (Hanganutziu, 1924; Deicher, 1926). The immunogenic target of HD antibodies was later identified as Neu5Gc-containing GM3 gangliosides (Nishimaki et al., 1979). Following on from this, these glycolipid antigens and other Neu5Gc-containing glycans were reported to accumulate on both foetal tissues and cancer cells, leading to their classification as oncofoetal antigens with potential diagnostic or therapeutic applications (Kawai et al., 1991; Muir et al., 1991). However, this view of Neu5Gc solely as a disease marker was later complicated by advances in techniques used to study sialylation, which enabled the identification of Neu5Gc-containing glycans, albeit at low levels, even in tissues from healthy adults (Padler-Karavani et al., 2008; Mastrangeli et al., 2021). This was particularly prevalent in tissues with high turnover rates, such as endothelial cells and epithelial surfaces (Tangvoranuntakul et al., 2003). This preferential accumulation of Neu5Gc at fast-growing, metabolically active surfaces may provide an explanation for the high levels of Neu5Gc-containing glycans reported in both foetal tissues and cancer cells (Wang et al., 2023). Growing appreciation for the incorporation of Neu5Gc into healthy tissues and the potential immunogenicity of these epitopes has prompted interest in the Neu5Gc content of glycosylated therapeutics such as monoclonal antibodies produced in non-human cells or bioprosthetics derived from mammalian tissues (Mastrangeli et al., 2021). For example, the monoclonal-antibody-based EGFR inhibitor cetuximab, produced in SP2/0 mouse myeloma cells, is estimated to contain Neu5Gc-glycoconjugates at multiple residues and has been reported to trigger an anti-Neu5Gc immune response in *Cmah* ^−/−^ mice (Ghaderi et al., 2010; Yehuda and Padler-Karavani, 2020). Similarly, treatment of type 1 diabetes patients with anti-thymocyte globulin (ATG) produced in rabbits was associated with an increase in both titres and diversity of anti-Neu5Gc antibodies (Le Berre et al., 2019), and burn patients treated with porcine skin grafts showed a long-term increase in anti-Neu5Gc antibodies compared to patients treated with conventional allografts (Scobie et al., 2013). This potential for Neu5Gc-containing biotherapeutics to trigger an immune response in humans has also led to strong interest in Neu5Gc in the field of xenotransplantation, where graft failure and rejection have been attributed to the induction of anti-graft immune responses against carbohydrate xenoantigens such as αGal and Neu5Gc in multiple studies (Blixt et al., 2009; Zhang et al., 2018; Chaban et al., 2022; Senage et al., 2022).

Along similar lines, anti-Neu5Gc antibody responses against Neu5Gc-containing glycans on human tissues have also been explored as a trigger of chronic inflammation-a process dubbed ‘xenosialitis’ (Dhar et al., 2019). *Cmah* ^−/−^ mice appear to be predisposed to a higher incidence of various conditions, such as diabetes-like insulin resistance and hearing loss (Okerblom and Varki, 2017). Similarly, exposing *Cmah* ^*−/−*^ *Ldlr* ^*−/−*^ mice to dietary Neu5Gc led to an increase in atherosclerosis-like lesions compared to mice fed a diet rich in Neu5Ac; however, it must be noted that this was only observed in mice directly immunised against Neu5Gc prior to dietary exposure (Kawanishi et al., 2021). This raises a key issue with the use of *Cmah* ^−/−^ mice in studies into the implications of Neu5Gc in human inflammatory disease. The induction of anti-Neu5Gc antibodies in this model does not occur spontaneously upon exposure and instead requires a strong, adjuvanted immunising event that may not be representative of dietary or microbiome-based induction events hypothesised to occur in early life in humans (Soulillou et al., 2020a). Additionally, *Cmah* deletion in these mice may not accurately represent the gradual evolutionary process of CMAH inactivation in the human population, which may be associated with various unexplored compensatory events (Okerblom and Varki, 2017). In consideration of this, *Cmah* ^−/−^ mice may be a more suitable model to investigate ‘elicited’ anti-Neu5Gc responses rather than the effects of pre-existing circulating anti-Neu5Gc antibodies in the general population (Soulillou et al., 2020b). Further complicating the field, studies in humans aiming to identify links between Neu5Gc and various inflammatory and autoimmune conditions, such as Kawasaki disease, Duchenne muscular dystrophy, and multiple sclerosis, frequently yield contradictory results (Padler-Karavani et al., 2013; Eleftheriou et al., 2014a; Le Berre et al., 2017; Boligan et al., 2020; Martin et al., 2021).

The use of murine models and conflicting results from population studies are similarly present in research investigating the hypothesised links between Neu5Gc and cancer. Anti-Neu5Gc antibodies have been theorised as a driver of tumour progression through stimulation of chronic antibody-mediated inflammation, supported to some extent by an increase in systemic inflammation markers such as IL-6 and acute phase proteins observed in *Cmah* ^−/−^ mice immunised against and fed Neu5Gc (Samraj et al., 2014). This inflammatory response was also linked to a slight increase in hepatocellular carcinoma incidence in these mice and was postulated as a potential contributor to the established association between red meat consumption and cancer incidence (Farvid et al., 2021). Similar results have also been reported from passive immunisation of tumour-bearing mice with serum positive for anti-Neu5Gc antibodies, which led to a slight increase in IgG antibody deposition, leucocyte infiltration, and endpoint tumour weight (Hedlund et al., 2008). Much of this research in mouse models is again not fully reflected in humans, with multiple large-scale studies reporting no clear association between titres of anti-Neu5Gc antibodies and cancer incidence in the general population (Samraj et al., 2018; Bashir et al., 2020).

### A complicated picture: the anti-Neu5Gc response in humans and the methods used to study it

Inconsistent, often contradictory findings are reflected across the whole field of research into the implications of Neu5Gc in human health and disease. Many studies suggest that immune responses against Neu5Gc may be a putative driver of cancer (Diccianni et al., 2018; Bashir et al., 2020), autoimmune disease (Eleftheriou et al., 2014a), inflammation (Pham et al., 2009), and degenerative conditions (Martin et al., 2021), with some even speculating that CMAH loss and resulting changes to the sialic acid landscape may have contributed to the increase in brain size and plasticity that allowed the development of human intelligence (Davies and Vark, 2013). Equally, many studies report no discernible link between disease and the presence of anti-Neu5Gc antibodies (Eleftheriou et al., 2014b; Le Berre et al., 2017; Soulillou et al., 2018), and some even propose that anti-Neu5Gc antibodies may have a protective role, particularly against allergic disease (Frei et al., 2018; Frei et al., 2019). Because of these discrepancies, the true extent of the anti-Neu5Gc antibody response in humans remains controversial. This inconsistency may reflect the fact that despite almost 50 years passing since Neu5Gc was identified as the main immunogenic component of the Hanganutziu–Deicher antigen in 1977 (Higashi et al., 1977), no standardised method for the detection or quantification of anti-Neu5Gc antibodies has been developed. While commercial test kits and established protocols are readily available for antibodies against other carbohydrate xenoantigens such as αGal (Buonomano et al., 1999; Lis et al., 2023), methods to detect anti-Neu5Gc antibodies in human serum range from flow cytometry to haemagglutination tests, to ELISA assays, to large-scale glycan arrays. This diversity makes it difficult to compare studies and, therefore, complicates any attempt to reach a consensus on the true prevalence of anti-Neu5Gc antibodies in humans. This is further impeded by the complexity of the anti-Neu5Gc humoral response, with anti-Neu5Gc antibodies described to accommodate Neu5Gc itself, as well as up to six units in its underlying structures (Breimer and Holgersson, 2019). This polyclonal response is often not considered in methods used to detect anti-Neu5Gc antibodies, with many utilising single homogenous epitopes that may fail to capture a significant portion of this complex immune response (Frei et al., 2019). Additionally, the ability of cells to uptake and incorporate Neu5Gc from exogenous sources also requires careful consideration, as mammalian serum frequently used in cell culture can skew the level of Neu5Gc on the cell surface and potentially impact methodologies using whole cells, such as flow cytometry (Martin et al., 2005; Villacrés et al., 2021). Along similar lines, mammalian proteins such as bovine serum albumin (BSA) are frequently used as blocking agents in standard protocols to detect serum antibodies, and endogenous Neu5Gc-containing glycans present on these proteins may interfere with the detection of anti-Neu5Gc antibodies.

Careful consideration of cell culture conditions, blocking agents, target epitopes, and many other factors is essential when exploring the implications of Neu5Gc in human health. Therefore, in this review, we aimed not only to generate a detailed exploration of how prevalent anti-Neu5Gc antibodies are in the human population but also to explore whether these reported values are influenced by the experimental design used. Our primary objective was to highlight experiment design considerations important to future research into the quantification of anti-Neu5Gc antibodies, and their controversial implications for human health, disease, and therapeutic interventions.

## Methods

Guidelines for Preferred Items for Systematic Reviews and Meta-Analysis (PRISMA 2020) were followed where appropriate.

### Registration and data availability

The study was not preregistered, as it did not include any health-related outcomes. Details of the study design are described herein, and tables of the data collected can be accessed in the Supplementary Material. Completed PRISMA checklists are also available in the Supplementary Material.

### Database search

Database searches were performed using the search terms listed in Table 1 using the PubMed, Scopus, and Web of Science databases, leading to identification of a total of 1,689 published articles. After automated removal of duplicates using EndNote 21 reference manager software (Clarivate, Philadelphia, PA), a total of 547 entries were forwarded for further screening.

**TABLE 1:** Databases screened and search terms used for the identification of initial entries.

Target database	Date of last search	Search terms	Number of entries identified
PubMed	14/11/23	((Neu5Gc) AND (Antibod*)) AND ((Human) OR (Serum))	116
		((N-glycolylneuraminic acid) AND (Antibod*)) AND ((Human) OR (Serum))	212
		(Hanganutziu-Deicher) AND (Antibod*) AND (Human)	63
Scopus	14/11/23	((Neu5Gc) AND (Antibod*)) AND ((Human) OR (Serum))	185
		((N-glycolylneuraminic acid) AND (Antibod*)) AND ((Human) OR (Serum))	237
		(Hanganutziu-Deicher) AND (Antibod*) AND (Human)	75
Web of Science	14/11/23	((ALL=(Neu5Gc)) AND ALL=(Antibod*)) AND ALL=(Human)	174
		((ALL=(Neu5Gc)) AND ALL=(Antibod*)) AND ALL=(Serum)	80
		((ALL=(N-glycolylneuraminic acid)) AND ALL=(Antibod*)) AND ALL=(Serum)	144
		((ALL=(N-glycolylneuraminic acid)) AND ALL=(Antibod*)) AND ALL=(human)	353

### Study selection and exclusion criteria

**FIGURE 2:**
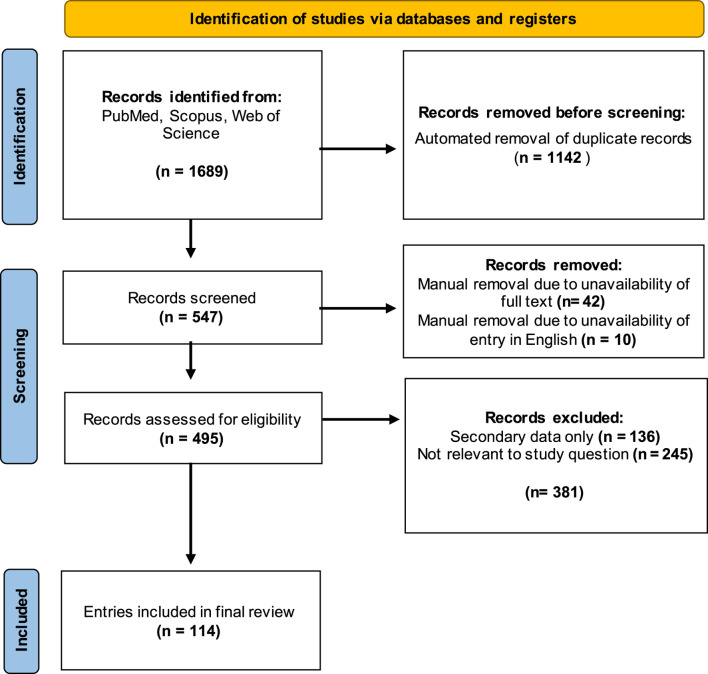
Outline of the selection process used to screen studies for the review. Three databases were queried, and 1,689 studies were identified using the specified search terms. After removing duplicates and manual screening for relevance, a final group of 114 articles was selected for inclusion. Template produced by PRISMA 2020.

Exclusion criteria were determined prior to screening to avoid bias in the selection of entries included in the final review and were not altered after the screening process began. Only entries presenting primary data were included, excluding literature reviews and opinion pieces (n = 136) from the collated entries. Entries that were not available in English were also excluded (n = 10). Articles where no full text was available, such as conference abstracts, were also removed (n = 11). Finally, articles were excluded on the basis of lack of relevance if they did not report attempts to quantify at least one class of human immunoglobulin directed against Neu5Gc or Neu5Gc-containing glycans (n = 245). Methods of detecting anti-Neu5Gc antibodies in non-human serum and methods to detect Neu5Gc-containing glycans in human tissues were, therefore, not included. After manual screening for relevance, the final list of entries included 114 articles published between 1977 and 2023. The screening process used for this review is outlined in Figure 2.

### Data collection

After entry identification, articles were screened for the information outlined in Table 2. The percentage of anti-Neu5Gc positive samples, average reported immunoglobulin titres, and inter-experiment variation were recorded as directly quoted in the text. If this information was not provided, then values were calculated or estimated from the data presented. Values were then grouped according to various experimental factors also outlined in Table 2.

**TABLE 2:** Outline of data collected for each review entry.

Data collected	Additional comments
Identifying information	N/A
Year published	Articles were not excluded based on date of publication, and publication date ranged from 1977–2023
Context	Biological context under which the primary sample group was studied. Entries were sorted into the categories of Healthy population, Xenotransplantation, Biotherapeutic use, Inflammation, and Infection and Cancer
Detailed context	Specific summary of the entry content
Sample number	Number of samples tested as part of the group of interest
Sample demographic	Demographic location from which serum or plasma samples were collected. Note that this indicates geographic location, not patient ethnicity
Sample preparation	Sample type (serum, plasma, *etc.*), storage, and heat inactivation
Detection method	Procedure used to assess anti-Neu5Gc antibodies. If multiple strategies were used, two separate entries were created
Target epitope	Neu5Gc-containing epitope used
Control epitope	Control epitopes used to determine non-specific signal or background reactivity
Target preparation	Method of epitope production. Epitopes isolated from an endogenous source, such as whole cells, glycoproteins, or gangliosides, are defined as ‘Natural.’ Epitopes produced using chemical syntheses, such as PAA-conjugated monosaccharides or synthetic glycans, are defined as ‘Synthetic’
Appropriate controls used	Entries with appropriate controls were defined as those where the control and target epitopes were matched, differing only by the presence of Neu5Gc or Neu5Ac
Blocking agent	Method used to block non-specific reactivity
Percentage of anti-Neu5Gc-positive samples	Percentage of total samples with anti-Neu5Gc reactivity higher than the signal reported from control epitopes. When multiple immunoglobulin classes were reported, IgG was used to determine anti-Neu5Gc positivity. Used to indicate assay sensitivity (recorded for 81 entries)
Antibody class	Immunoglobulin classes investigated
Antibody titre	Mean reported value for each immunoglobulin class, as appropriate. Recorded using the units described in the entry (not adjusted). Only data from entries assessing exact antibody titres using an immunoglobulin standard curve was reported (22 entries)
Variation	Standard deviation, standard error, or range of values reported
Adjusted variation	Variation in reported titres, adjusted as a proportion of the average reported anti-Neu5Gc value. *Adjusted variation = (Reported variation/mean value) × 100*. Used to indicate inter-assay variability. Only data from entries where variation was reported as standard deviation or standard error was included in the final report (37 entries)

Review data were processed using RStudio (RStudio Team, RStudio: Integrated Development for R, PBC, Boston, MA) and analysed and presented using GraphPad Prism Version 9.4.1 (Dotmatics, United States of America). Data are presented to show median values, the 25th and 75th percentiles, and the minimum and maximum values, unless stated otherwise.

## Results and discussion

### The landscape of current research into anti-Neu5Gc antibodies

**FIGURE 3:**
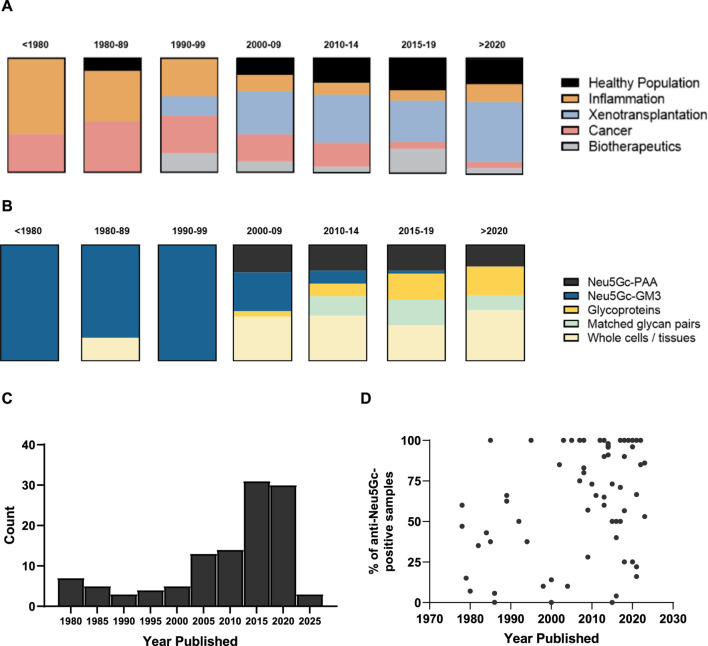
Chronological overview of research into anti-Neu5Gc antibodies over time. **(A + B)** Graphical representation of the context **(A)** and target epitope of choice **(B)** in studies into anti-Neu5Gc antibodies in humans, grouped by year. Blocks represent the total number of entries for each year bracket (n= 3, 10, 5, 21, 18, 36, and 16, respectively). PAA, polyacrylamide. **(C)** Histogram displaying the number of articles published in each year bracket. **(D)** Scatter plot of the percentage of reported anti-Neu5Gc antibody positive samples plotted by year published. Each point is representative of one entry.

Starting with the identification of Neu5Gc as the immunogenic component of the HD antigen in the late 1970s (Nishimaki et al., 1979), the present review identified 114 published articles presenting primary data on the detection and quantification of anti-Neu5Gc antibodies in humans, spanning a period of 45 years. Early research into anti-Neu5Gc antibodies focused strongly on their hypothesised role in inflammation, mainly exploring links between ‘heterophile antibodies’ against Neu5Gc-containing ganglioside GM3 (the ‘HD antigen’) and inflammatory conditions such as Kawasaki disease, rheumatoid arthritis, and motor neurone disease (Nishimaki, 1979; Arita et al., 1982; Yoshino et al., 1992). As interest in Neu5Gc-GM3 as a putative tumour biomarker and therapeutic target expanded, so did the field of research around this topic, with multiple studies exploring the induction of serum antibodies against Neu5Gc-GM3 following treatment with anti-idiotypic monoclonal antibodies against this epitope. For example, 1E10 underwent clinical trials in breast cancer and non-small cell lung cancer patients in the early-to mid-2000s (Díaz et al., 2003; Hernández et al., 2008) (Figure 3A). The specific focus on the HD antigen as a tumour marker and driver of xenosialitis meant that most early assays investigating serum antibodies against Neu5Gc used Neu5Gc-GM3 glycolipids as target epitopes (Figure 3B). These ganglioside antigens were typically isolated from bovine erythrocyte membranes and may have only accounted for a small portion of the total polyclonal anti-Neu5Gc response.

In the early 2000s, the advent of xenotransplantation marked a notable shift in the focus of studies into anti-Neu5Gc antibodies (Padler-Karavani and Varki, 2011). Neu5Gc was hypothesised as a non-αGal antigen responsible for xenograft rejection of α1,3-galactosyltransferase-knockout porcine tissues (Kobayashi et al., 2000; Diswall et al., 2007). This interest in anti-Neu5Gc antibodies in the context of xenotransplantation was marked by an increase in the use of flow cytometry to detect human serum antibody binding to whole porcine cells or homogenised tissues, often following deletion of the *Cmah* gene (Lutz et al., 2013) (Figure 3B). While the diverse array of Neu5Gc-containing glycans presented on cells is likely more analogous to how these glycans would be arranged in an endogenous context, these flow cytometry-based assays often gave only an indirect indication of the presence of anti-Neu5Gc antibodies in human serum and were not suitable to quantify exact antibody titres.

Alongside the interest in Neu5Gc as a potential confounding factor for the clinical application of xenografts and biotherapeutics, the late 2000s also marked an increase in the number of studies focussing on anti-Neu5Gc antibodies in the general population (Figure 3A). This perhaps coincided with the development of newer, more sensitive, and, importantly, higher throughput methods of detecting these antibodies in human serum, such as the glycan microarray (Padler-Karavani et al., 2011), and the utilisation of serum glycoproteins from wild-type (WT) and *Cmah* ^−/−^ mice as a source of diverse Neu5Gc-positive and Neu5Gc-negative epitopes, first utilised in an ELISA protocol developed by Padler-Karavani et al. (2013). These strategies had the benefit of both representing a diverse range of Neu5Gc-containing glycans suited to the polyclonal nature of the anti-Neu5Gc response in humans, like the use of whole cells and tissues in flow cytometry, while also holding the potential for quantitative assessment of anti-Neu5Gc antibody titres.

This growing appreciation for the complexity of the anti-Neu5Gc antibody response and its potential relevance to multiple aspects of human health was reflected by a diversification in both the areas of human biology covered by the field of research and the epitopes targeted within these studies. Importantly, a positive association could be seen when the reported prevalence of anti-Neu5Gc antibodies was plotted over time (Figure 3D), which is likely a reflection of the increasing number of investigations into anti-Neu5Gc antibodies.

**FIGURE 4:**
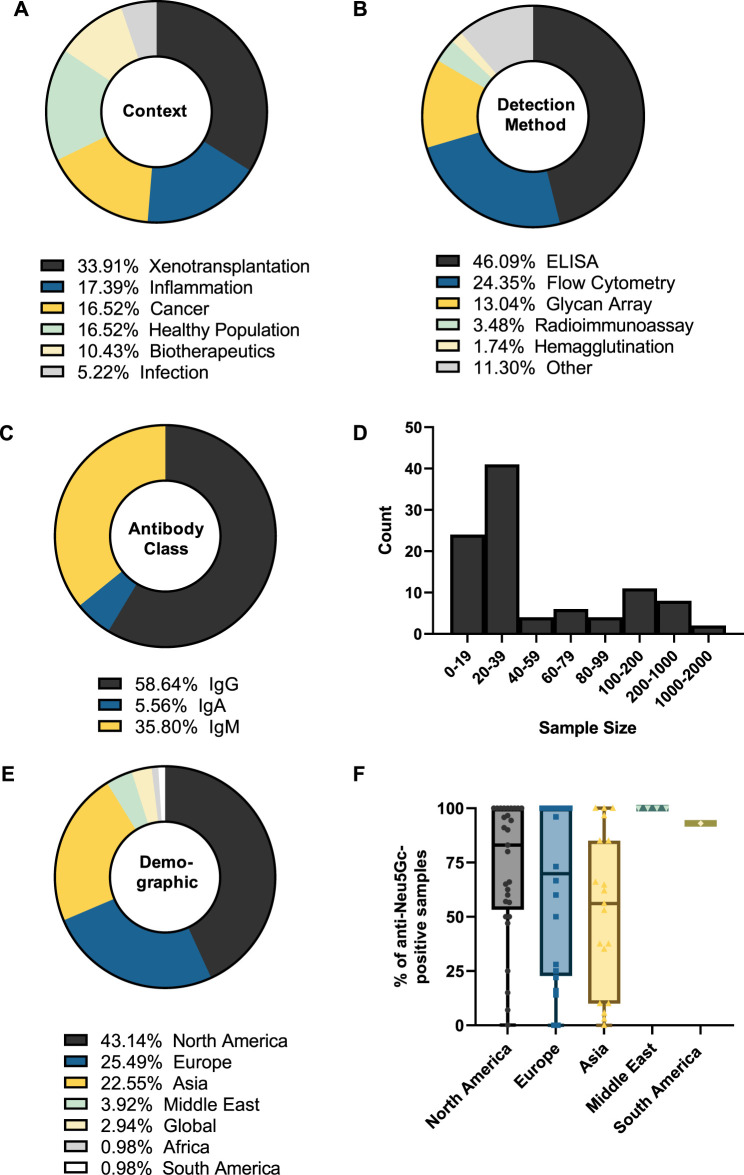
Summary of the features of studies into anti-Neu5Gc antibodies in the human population. The biological context **(A)**, detection method **(B)**, antibody class recorded **(C)**, sample size **(D),** and sample demographic **(E)** of 114 entries were recorded. Values represent the percentage of total entries. In **(B),** categories with two or fewer entries were recorded as ‘Other’. **(F)** The percentage of samples reported as anti-Neu5Gc positive grouped by sample demographic.

Analysis of the 114 studies published between 1977 and 2023 also revealed that xenotransplantation research accounts for around one-third of the literature focused on the humoral immune response against Neu5Gc (Figure 4A). Over half of these studies (54%) utilised flow cytometry to assess the binding of human immunoglobulins to various epitopes, from genetically modified pig cells to bioprosthetic heart valves and to embryonic stem cells grown on mammalian feeder cells, all with the overall goal of understanding and preventing the mechanisms behind graft rejection (Martin et al., 2005; Lee et al., 2016; Wang et al., 2019). Equal attention has been paid to potential roles for anti-Neu5Gc antibodies in inflammatory disease, cancer, and in the general population (Figure 4A). These studies utilised a diverse range of detection methods, with earlier approaches using haemagglutination tests and radioimmunoassays to explore the accumulation of heterophile HD antibodies on Neu5Gc-positive bovine erythrocytes (Higashi et al., 1977), and more modern approaches using high-throughput methods such as glycan microarrays to screen large populations for antibodies against a wide range of Neu5Gc-containing glycans (Ben-Arye et al., 2017) (Figure 4B). However, diversification of the methods used to detect anti-Neu5Gc antibodies in human serum is not without issue, leading to significant difficulties when aiming to compare the values reported across different studies. This lack of a standardised approach for anti-Neu5Gc antibodies is likely a major factor in why the true extent of this response is still a subject of much debate.

Anti-carbohydrate antibodies typically fall under the IgM and IgG subclasses, hence the bias towards these isotypes in the entries covered by the review, together accounting for around 95% of the included studies (Figure 4C). Only a small portion of studies investigated the prevalence of IgA class antibodies against Neu5Gc. This may indicate an important knowledge gap, as IgA antibodies are typically associated with mucosal surfaces, such as the digestive tract, where initial exposure to dietary Neu5Gc is likely to occur (Byres et al., 2008). A similar gap can be seen in the lack of studies investigating the individual subtypes of anti-Neu5Gc IgG antibodies. Anti-carbohydrate antibodies are often thought to predominantly belong to the IgG_2_ subclass. Immunoglobulins of this subclass are associated with a limited ability to trigger downstream effector responses, such as antibody-dependent cellular cytotoxicity or complement deposition, which is typically the product of an IgG_1_-or IgG_3_-mediated response (Vidarsson et al., 2014; Kappler and Hennet, 2020). However, anti-carbohydrate responses encompassing all IgG subclasses have been reported (Kappler and Hennet, 2020), and the small number of studies assessing specific subtypes of anti-Neu5Gc IgG reported a high prevalence of IgG_1_ subclass antibodies mounted against Neu5Gc (Saethre et al., 2006; Lu et al., 2012; Hurh et al., 2016). Therefore, further understanding of this area may provide important insights into the downstream effector functions of the anti-Neu5Gc response.

This overview of collected entries also revealed that most studies utilised sample sizes of fewer than 40 participants, with a median sample size of 20 individuals (Figure 4D). While large-scale population studies (>500 participants) have been attempted, these were few (n = 5). As the anti-Neu5Gc antibody response is polyclonal and affected by a range of exogenous factors, such as the diet and microbiome, it is likely to exhibit strong individual variation (Dhar et al., 2019). Because of this variation, these large-scale population studies may ultimately be essential to achieve adequate power to accurately identify patterns in the anti-Neu5Gc immune response.

Grouping studies by sample demographic (Figure 4E) revealed that most samples were collected from Western countries, with studies in Europe and North America accounting for around two-thirds of the collected data. Plotting the percentage of anti-Neu5Gc antibody-positive samples according to these different populations (Figure 4F) revealed potential trends in the prevalence of anti-Neu5Gc antibodies across different demographics, which may be linked to exogenous factors such as the amount of red meat or dairy consumed in the diet. These hypothetical links warrant further investigation, which cannot be achieved without first addressing the bias towards Western countries in existing data.

### Quantity of anti-Neu5Gc antibodies in the human population

**FIGURE 5:**
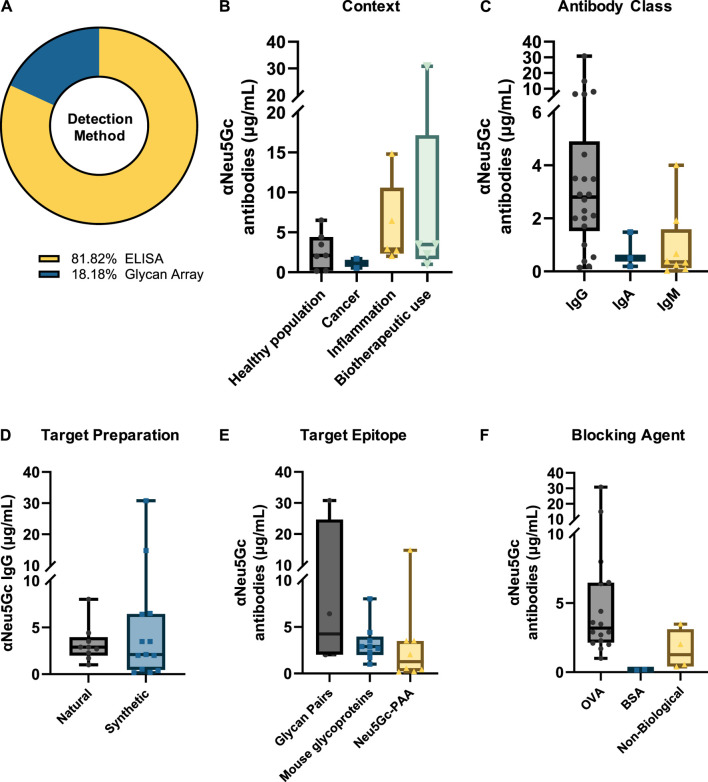
Summary of variation in absolute titres of anti-Neu5Gc antibodies. **(A)** Absolute values for the serum concentration of anti-Neu5Gc antibodies were recorded in 22 ELISA and glycan array studies. Titres were grouped according to immunoglobulin isotype **(B)**, study context **(C)**, epitope preparation **(D),** target epitope **(E),** and blocking agent used **(F)** to explore both biological and intra-experimental variation in anti-Neu5Gc antibody concentration. In all graphs, boxes represent the 25th–75th percentiles, with intersecting lines indicating the median values. Whiskers indicate the maximum and minimum values.

Absolute values for the concentration of anti-Neu5Gc antibodies in human serum were reported in 22 of the 114 entries. Most of these studies used an ELISA approach (81.82%), with the remaining utilising glycan microarrays (Figure 5A).

Reported titres exhibited substantial variation between experiments across all isotypes studied. Anti-Neu5Gc IgG concentration was reported to be the highest, with a median value of 2.80 μg/mL, although this ranged from 0.15 to 30.80 μg/mL (Figure 5B). IgA was reported at a median value of 0.5 μg/mL, and IgM titres were the lowest, reported at a median of 0.35 μg/mL. This is consistent with the proportion of antibody isotypes described in normal human serum, with IgG, IgA, and IgM accounting for 75, 15%, and 10% of the total immunoglobulin pool, respectively (Schroeder and Cavacini, 2010).

When grouped according to context, the highest titres of anti-Neu5Gc antibodies were reported in studies investigating the use of Neu5Gc-containing biotherapeutics, with a median value of 3.48 μg/mL, compared to 2.1 μg/mL in the healthy population (Figure 5C). Importantly, in some of these biotherapeutics studies, phenotypic differences in the anti-Neu5Gc antibody response were reported alongside an increase in overall titres following treatment. For example, anti-Neu5Gc IgG affinity-purified from type 1 diabetes patients treated with anti-thymocyte globulin (ATG) produced in rabbits showed a repertoire shift between pre-existing and elicited antibodies on a glycan array and exerted differential effects on endothelial cell activation (Le Berre et al., 2019). This increase in circulating antibodies and phenotypic changes following exposure to high levels of Neu5Gc is consistent with the kinetics of a secondary immune response and may be indicative of immunological memory against Neu5Gc in at least some portion of the human population (Soulillou et al., 2020a).

Another key observation from this analysis is a notable variation between different experiments, influenced by various conditions such as the choice of target epitope (Figure 5E) and blocking agent (Figure 5F). For example, assays utilising an array of matched Neu5G/Ac-containing glycan pairs reported higher median titres (4.25 μg/mL) than those using monomeric Neu5Gc-Polyacrylamide (PAA) (1.27 μg/mL), perhaps highlighting that a diverse range of epitopes is more suited to capturing the full extent of the polyclonal anti-Neu5Gc antibody response. Importantly, these changes could not be accounted for by differences in the method of epitope preparation – for example, whether Neu5Gc-containing glycans were obtained from natural glycoproteins or chemically synthesised– which did not appear to affect median reported antibody titres (Figure 5D). Furthermore, even within the same context, extensive variation in reported anti-Neu5Gc antibody titres can be seen between studies. While biotherapeutic exposure yielded the highest median titres of anti-Neu5Gc antibodies, as previously discussed, these values ranged from 1.0 to 30.8 μg/mL and were likely influenced by the specific experimental conditions used. This intra-experimental variation aligns well with previous evidence that different glycan array preparation methods strongly influenced the binding activity of various sialic acid binding proteins, such as SNA and MAL II lectins, Siglecs, and both polyclonal and monoclonal IgY antibodies against Neu5Gc (Padler-Karavani et al., 2012). This finding reinforces the need for a standardised approach to the detection and quantification of anti-Neu5Gc antibodies in human serum if genuine and comparable patterns in antibody titres are to be identified.

### Prevalence of anti-Neu5Gc antibodies in the human population

As only a small portion of the studies covered reported exact anti-Neu5Gc antibody titres in human serum, the percentage of samples reported as ‘anti-Neu5Gc antibody positive’ (determined as the proportion of samples with values exceeding the background level) was also recorded to allow comparison between a wider range of studies. This percentage-positive value may be used to infer the prevalence of anti-Neu5Gc antibodies in the population studied and may also be indicative of the sensitivity of the assay used to detect these antibodies. Only the IgG isotype value was plotted for these entries to avoid overrepresenting studies where multiple immunoglobulin isotypes were recorded. Positive rates for additional isotypes are recorded in the Supplementary Material.

**FIGURE 6:**
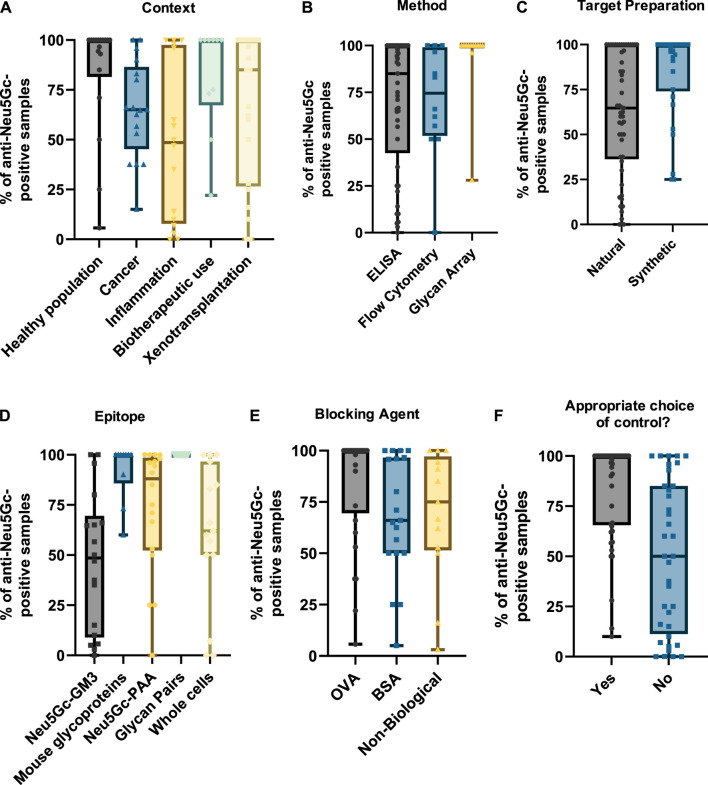
Graphs summarising the percentage of anti-Neu5Gc-positive samples. Data were collected from 81 entries and grouped by context **(A)**, detection method **(B)**, epitope preparation **(C)**, target epitope **(D)**, blocking agent **(E),** and whether appropriate controls were used—defined as the use of matched Neu5Gc- and Neu5Ac-containing epitopes **(F)**. Only IgG values were plotted in studies where multiple immunoglobulin isotypes were reported to avoid overrepresentation of entries. In all graphs, boxes represent the interquartile range, with intersecting lines indicating the median values. Whiskers indicate the maximum and minimum values.

As in the previous section, grouping studies by context (Figure 6A) indicated that biotherapeutic use led to the highest median value, with most studies reporting that anti-Neu5Gc antibodies were present in all samples investigated. Interestingly, this was matched by the healthy population, which also reported most samples as anti-Neu5Gc positive. Studies focussing on the prevalence of anti-Neu5Gc antibodies in cancer patients and in the context of inflammation and xenotransplantation revealed a much higher level of variation in the reported prevalence of anti-Neu5Gc antibodies, with some studies reporting a complete absence of anti-Neu5Gc antibodies, while others found that these antibodies were ubiquitous across the participants investigated.

While the prevalence of anti-Neu5Gc antibodies would be expected to differ across various populations due to the influence of diet, microbiome composition, and many other factors, the extent of the variation reported across these studies is unlikely to be solely biological. Grouping entries by method (Figure 6B) and by target epitope (Figure 6D) shows a trend towards a higher positive rate in assays utilising a diverse array of epitopes, such as glycan arrays and ELISAs coated with Neu5Gc-containing glycoproteins from *Cmah*-positive WT mice with median positive rates of 100% in both conditions. Importantly, these sensitive strategies present Neu5Gc in the context of a range of underlying structures analogous to its appearance in the human body. These were preferentially utilised in the aforementioned studies investigating biotherapeutics and the healthy population. In comparison, studies into inflammation and cancer strongly favour ELISA tests against a single Neu5Gc-containing epitope, the Hanganutziu–Deicher antigen. This is probably not suited to detect the full range of anti-Neu5Gc antibodies in humans and may explain a median positive rate of only 48.5% (Figure 6D). This outcome could also be seen when studies were grouped according to the general method of target epitope preparation (Figure 6C). The trend towards lower percentage positive rates in ‘naturally’ prepared Neu5Gc-containing glycans was likely skewed by the abundance of studies in this group utilising the HD antigen, which is typically isolated directly from bovine or equine erythrocytes.

A similar effect could be seen when entries were grouped by choice of blocking agent (Figure 6E). Indeed, using proteins from mammals endogenously synthesising Neu5Gc like BSA or using ovalbumin (OVA) from chickens lacking Neu5Gc synthesis seems to impact the proportion of anti-Neu5Gc antibody-positive samples (66% and 100%, respectively). A potential explanation for this trend is the presence of Neu5Gc on glycans at the surface of these proteins that may ‘mop up’ anti-Neu5Gc antibodies, reducing detection rates. Another factor that may further influence the reported prevalence of anti-Neu5Gc antibodies is the choice of control epitope. When detecting antibodies in human serum, a highly complex mixture of proteins, glycans, and immunoglobulins, appropriate controls are essential to distinguish true signals from background noise generated by non-specific reactivity. This is especially important when presenting an array of targets rather than a single epitope, as this complexity increases the likelihood of recognition of other epitopes by serum antibodies. In these cases, matched pairs of target and control epitopes containing Neu5Gc and Neu5Ac, respectively, are ideal because they only differ by a single hydroxyl group responsible for immunogenicity. However, half of the studies included did not utilise these appropriately matched pair controls. This may have an impact on the ability of these studies to obtain a true value for the prevalence of anti-Neu5Gc antibodies in the target population, highlighted by a trend in reduced average positive rates compared to studies where matched pairs were included (50.1% and 83.7%, respectively) (Figure 6F).

This potential for experimental factors to notably affect the reported prevalence of anti-Neu5Gc antibodies in the general population may also explain the trend towards an overall higher positive rate in more modern studies (Figure 3D), with assays becoming more sensitive over time. However, this also raises issues for the interpretation of these findings, with sample preparation, detection method, epitope choice, and many other experimental factors potentially masking the true prevalence of anti-Neu5Gc antibodies in humans. Patterns in the reported incidence of anti-Neu5Gc antibodies in various human populations may, therefore, not be due to biological differences and may instead be a consequence of experimental design. Comparisons between studies, even those investigating the same population, should be made with caution.

### Individual variation in the anti-Neu5Gc antibody response

**FIGURE 7:**
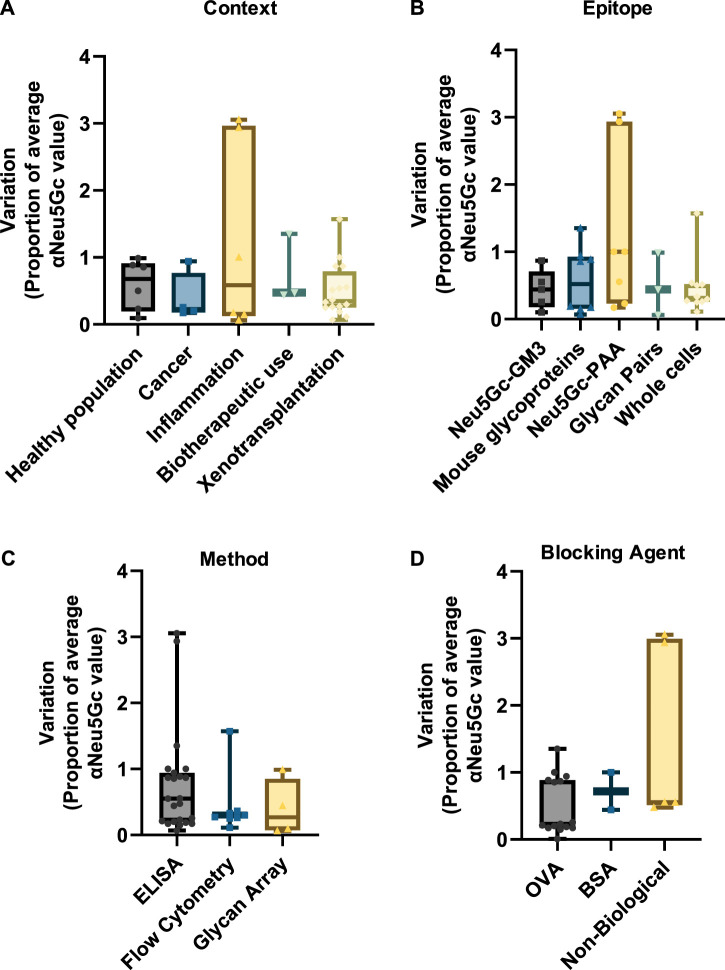
Graphs indicating the inter-experimental variation in assays to detect anti-Neu5Gc antibodies. Standard deviation values from 37 entries were normalised as a proportion of the average reported anti-Neu5Gc antibody titre. Entries were grouped according to context **(A)**, target epitope **(B)**, detection method **(C),** and blocking agent **(D)**. Boxes cover the interquartile range of the data, with intersecting bars indicating the median value. Whiskers show the maximum and minimum values. Studies where variation was reported as a range instead of standard deviation were not included.

As highlighted *vide supra*, research into anti-Neu5Gc antibodies in humans is subject to extensive variation between studies. However, a similar degree of variation can also be observed within experiments. This is likely corresponding to individual differences in anti-Neu5Gc antibodies across the population. Variation was normalised as a proportion of the average reported value for anti-Neu5Gc antibodies in the population studied to allow for comparison between a wider range of studies within the review and account for the different units used across different detection strategies. Only entries where standard deviation or standard error was directly stated were included in this analysis. In entries where this information was not reported, the range between the highest and lowest reported values was used to indicate variation. These values are recorded in the Supplementary Material. Unlike the reported prevalence or absolute titres of anti-Neu5Gc antibodies, inter-experiment variation was relatively consistent across different contexts (Figure 7A), with median values falling between 0.28 (cancer) and 0.68 (healthy population).

Similar amounts of variation were also seen across different experimental factors, such as the target epitope (Figure 7B), the detection method (Figure 7C), and the choice of blocking agent (Figure 7D). While some entries reported unusually high levels of variation between samples [notably, two studies investigating links between anti-Neu5Gc antibodies and allergic disease in children from rural environments (Frei et al., 2018; Frei et al., 2019)], the overall consistency of this inter-experiment variation suggests a genuine biological trend towards high individual variation in anti-Neu5Gc antibody titres. This agrees with the current literature, which suggests that anti-Neu5Gc antibodies may vary according to diet, sex, demographic, exposure to microorganisms, vaccination history, and other factors, many of which are likely to interact in a complex network, conceivably resulting in a significant amount of individual variation (Taylor et al., 2010; Gao et al., 2017; Samraj et al., 2018; Bashir et al., 2020; Martin et al., 2021). As a consequence of this variation, large sample sizes are likely to be essential in achieving the experimental power required to identify genuine patterns and trends linking specific factors to anti-Neu5Gc antibodies in humans.

## Conclusion

Whilst efforts were made to explore the full scope of the literature focussing on the anti-Neu5Gc immune response in humans, limitations in the design of this review must be noted. The challenge in making comparisons between different methodologies meant that data had to be adjusted in some cases, for example, through normalisation of variation as a proportion of the average. Similarly, quantitative information had to be estimated from graphical representations in studies where this was not directly stated in the text, potentially introducing errors. In order to include xenotransplantation studies in the review, which accounted for over half of the literature focused on the anti-Neu5Gc humoral response, a decision was also made to include studies investigating non-αGal antibodies in GGTA knockout porcine tissues, which may have encompassed other xenoantigens aside from Neu5Gc.

Despite these limitations, this review highlighted a notable shift in the trajectory of research into the role of Neu5Gc and antibodies against it in human health over time, expanding from a field primarily interested in the induction of serum sickness and inflammation following exposure to the HD antigen to exploring a broad array of implications for therapeutic development, cancer, xenotransplantation, and autoimmune disease. Whilst this diversification represents a more nuanced, deeper understanding of the complex role that Neu5Gc may play in humans, it also presents significant complications when trying to reach a consensus on the extent of its impact, leaving the field a place of much confusion and conflicting evidence. As a result, a true value for the extent of the anti-Neu5Gc antibody response cannot be inferred from these studies.

This review also highlighted that individual variation cannot be ignored when considering the prevalence of anti-Neu5Gc antibodies in the general population. As a unique xenoautoantigen, exposure to Neu5Gc in humans is dependent on extrinsic factors- such as the diet, the microbiome, previous infections, and medical history (Tangvoranuntakul et al., 2003; Taylor et al., 2010; Scobie et al., 2013; Le Berre et al., 2017)- as well as endogenous processes such as the overall rate of sialic acid turnover (Yin et al., 2006). Logically, this led to extensive individual variation within most studies covered by the review. The influence of a range of factors on the prevalence of anti-Neu5Gc antibodies may also explain why studies failed to establish links between single factors such as diet and antibody titres and why antibodies against Neu5Gc have been described even in individuals with limited red meat consumption (Gao et al., 2017; Bashir et al., 2020). To account for this variation, studies aiming to establish an association between specific factors and the prevalence of anti-Neu5Gc antibodies will likely require large sample sizes to achieve adequate power. Where this is not achievable, it may also be possible to exploit pooled samples, such as commercial serum or therapeutic intravenous immunoglobulin (IVIG) that are collected from a wide range of donors and may be suitable to represent a population average.

Notably, this review identified important gaps in current knowledge about antibodies against Neu5Gc and suggested potential directions for further investigation. Specifically, assessing the prevalence of non-IgG antibodies against Neu5Gc may provide important additional information about the kinetics and consequences of the immune response towards Neu5Gc. This is particularly important for IgA, the prevalence of which was only explored in nine of the 114 studies, as this isotype is active at mucosal surfaces, where dietary absorption of Neu5Gc is predicted to occur. To a similar end, further exploration of IgG subtypes mounted against Neu5Gc may provide important information about the downstream implications of generating an immune response against this antigen, which will be especially relevant to its hypothesised role in human disease or, indeed, as a therapeutic avenue. In addition, very few studies reported exact values for the titres of anti-Neu5Gc antibodies, further preventing a consensus value to use as a standard from being reached.

Overall, this review highlighted that appropriate conclusions cannot be currently made about the prevalence of anti-Neu5Gc antibodies as it is not possible to ascribe trends and differences to genuine biological effects or the consequence of sample preparation and experiment design. Ultimately, this reinforces the need to establish a standardised method to study anti-Neu5Gc antibodies. Ideally, this would utilise a diverse array of Neu5Gc-containing epitopes to account for the polyclonal nature of the anti-Neu5Gc response, avoid the use of Neu5Gc-containing mammalian products in sample preparation, utilise a ‘matched pair’ system where target and control epitopes differ only by the presence of Neu5Gc or Neu5Ac respectively, and provide the option for scalability to population studies with large sample sizes.

As it currently stands, our understanding of the immune response against Neu5Gc is limited by differing methods used to assess antibody titres, the utilisation of inappropriate controls, and the lack of large-scale studies to account for individual variation. These factors are all likely to account for the conflicting evidence for the prevalence of antibodies against Neu5Gc. Furthermore, this lack of a consensus value raises an important issue for the translational application of research into Neu5Gc. The anti-Neu5Gc antibody response is described to follow an inverse hormesis model, in which low antibody titres may stimulate chronic low-level inflammation, and high titres stimulate immune clearance mechanisms such as antibody-dependent cellular cytotoxicity (Pearce et al., 2014). This dose-dependency is especially relevant for attempts to exploit the increased presence of Neu5Gc on tumour tissues as a therapeutic target (Machado et al., 2011) or in efforts to develop new biotherapeutic products with reduced risk of rejection (Komoda et al., 2010; Chaban et al., 2022) as the outcome of this would depend heavily on the titres of circulating antibodies present in each individual. Therefore, an inability to agree on the extent of the anti-Neu5Gc antibody response both raises questions about the relevance of this unique xenoautoantigen to human health and presents an important roadblock for these areas of research.
