# Supplementary material for: A systematic review reveals conflicting evidence for the prevalence of antibodies against the sialic acid ‘xenoautoantigen’ Neu5Gc in humans and the need for a standardised approach to quantification
Source: Front Mol Biosci. 2024 Apr 26;11:1390711. doi: 10.3389/fmolb.2024.1390711 (PMC11082328; doi:10.3389/fmolb.2024.1390711)
Supplement: Supplementary file 1 [file DataSheet1.pdf]

Supplementary Material

1- PRISMA checklist

Section and Topic	Item #	Checklist item	Reported (Yes/No)
**TITLE**			
Title	1	Identify the report as a systematic review.	Yes
**BACKGROUND**			
Objectives	2	Provide an explicit statement of the main objective(s) or question(s) the review addresses.	Yes
**METHODS**			
Eligibility criteria	3	Specify the inclusion and exclusion criteria for the review.	Yes
Information sources	4	Specify the information sources (e.g. databases, registers) used to identify studies and the date when each was last searched.	Yes
Risk of bias	5	Specify the methods used to assess risk of bias in the included studies.	N/A
Synthesis of results	6	Specify the methods used to present and synthesise results.	Yes
**RESULTS**			
Included studies	7	Give the total number of included studies and participants and summarise relevant characteristics of studies.	Yes
Synthesis of results	8	Present results for main outcomes, preferably indicating the number of included studies and participants for each. If meta-analysis was done, report the summary estimate and confidence/credible interval. If comparing groups, indicate the direction of the effect (i.e. which group is favoured).	Yes
**DISCUSSION**			
Limitations of evidence	9	Provide a brief summary of the limitations of the evidence included in the review (e.g. study risk of bias, inconsistency and imprecision).	No
Interpretation	10	Provide a general interpretation of the results and important implications.	Yes
**OTHER**			
Funding	11	Specify the primary source of funding for the review.	Yes
Registration	12	Provide the register name and registration number.	N/A

Section and Topic	Item #	Checklist item	Location where item is reported
**TITLE**			
Title	1	Identify the report as a systematic review.	Title
**ABSTRACT**			
Abstract	2	See the PRISMA 2020 for Abstracts checklist.	See Abstract checklist
**INTRODUCTION**			
Rationale	3	Describe the rationale for the review in the context of existing knowledge.	Introduction
Objectives	4	Provide an explicit statement of the objective(s) or question(s) the review addresses.	Introduction
**METHODS**			
Eligibility criteria	5	Specify the inclusion and exclusion criteria for the review and how studies were grouped for the syntheses.	Methods
Information sources	6	Specify all databases, registers, websites, organisations, reference lists and other sources searched or consulted to identify studies. Specify the date when each source was last searched or consulted.	Table 1
Search strategy	7	Present the full search strategies for all databases, registers and websites, including any filters and limits used.	Table 1
Selection process	8	Specify the methods used to decide whether a study met the inclusion criteria of the review, including how many reviewers screened each record and each report retrieved, whether they worked independently, and if applicable, details of automation tools used in the process.	Methods
Data collection process	9	Specify the methods used to collect data from reports, including how many reviewers collected data from each report, whether they worked independently, any processes for obtaining or confirming data from study investigators, and if applicable, details of automation tools used in the process.	Methods
Data items	10a	List and define all outcomes for which data were sought. Specify whether all results that were compatible with each outcome domain in each study were sought (e.g. for all measures, time points, analyses), and if not, the methods used to decide which results to collect.	Table 2
	10b	List and define all other variables for which data were sought (e.g. participant and intervention characteristics, funding sources). Describe any assumptions made about any missing or unclear information.	Table 2
Study risk of bias assessment	11	Specify the methods used to assess risk of bias in the included studies, including details of the tool(s) used, how many reviewers assessed each study and whether they worked independently, and if applicable, details of automation tools used in the process.	N/A
Effect measures	12	Specify for each outcome the effect measure(s) (e.g. risk ratio, mean difference) used in the synthesis or presentation of results.	Methods
Synthesis methods	13a	Describe the processes used to decide which studies were eligible for each synthesis (e.g. tabulating the study intervention characteristics and comparing against the planned groups for each synthesis (item #5)).	Figure 2
	13b	Describe any methods required to prepare the data for presentation or synthesis, such as handling of missing summary statistics, or data conversions.	Methods
	13c	Describe any methods used to tabulate or visually display results of individual studies and syntheses.	Methods

Section and Topic	Item #	Checklist item	Location where item is reported
	13d	Describe any methods used to synthesize results and provide a rationale for the choice(s). If meta-analysis was performed, describe the model(s), method(s) to identify the presence and extent of statistical heterogeneity, and software package(s) used.	Methods
	13e	Describe any methods used to explore possible causes of heterogeneity among study results (e.g. subgroup analysis, meta-regression).	Methods
	13f	Describe any sensitivity analyses conducted to assess robustness of the synthesized results.	N/A
Reporting bias assessment	14	Describe any methods used to assess risk of bias due to missing results in a synthesis (arising from reporting biases).	N/A
Certainty assessment	15	Describe any methods used to assess certainty (or confidence) in the body of evidence for an outcome.	N/A
**RESULTS**			
Study selection	16a	Describe the results of the search and selection process, from the number of records identified in the search to the number of studies included in the review, ideally using a flow diagram.	Figure 2
	16b	Cite studies that might appear to meet the inclusion criteria, but which were excluded, and explain why they were excluded.	N/A
Study characteristics	17	Cite each included study and present its characteristics.	Supplementary Materials
Risk of bias in studies	18	Present assessments of risk of bias for each included study.	N/A
Results of individual studies	19	For all outcomes, present, for each study: (a) summary statistics for each group (where appropriate) and (b) an effect estimate and its precision (e.g. confidence/credible interval), ideally using structured tables or plots.	Supplementary Materials
Results of syntheses	20a	For each synthesis, briefly summarise the characteristics and risk of bias among contributing studies.	N/A
	20b	Present results of all statistical syntheses conducted. If meta-analysis was done, present for each the summary estimate and its precision (e.g. confidence/credible interval) and measures of statistical heterogeneity. If comparing groups, describe the direction of the effect.	Figures 3, 4, 5, 6 and 7
	20c	Present results of all investigations of possible causes of heterogeneity among study results.	Results
	20d	Present results of all sensitivity analyses conducted to assess the robustness of the synthesized results.	N/A
Reporting biases	21	Present assessments of risk of bias due to missing results (arising from reporting biases) for each synthesis assessed.	N/A
Certainty of evidence	22	Present assessments of certainty (or confidence) in the body of evidence for each outcome assessed.	N/A
**DISCUSSION**			
Discussion	23a	Provide a general interpretation of the results in the context of other evidence.	Conclusion
	23b	Discuss any limitations of the evidence included in the review.	Conclusion
	23c	Discuss any limitations of the review processes used.	Conclusion
	23d	Discuss implications of the results for practice, policy, and future research.	Conclusion

Section and Topic	Item #	Checklist item	Location where item is reported
**OTHER INFORMATION**			
Registration and protocol	24a	Provide registration information for the review, including register name and registration number, or state that the review was not registered.	Not registered
	24b	Indicate where the review protocol can be accessed, or state that a protocol was not prepared.	Supplementary Materials
	24c	Describe and explain any amendments to information provided at registration or in the protocol.	N/A
Support	25	Describe sources of financial or non-financial support for the review, and the role of the funders or sponsors in the review.	Acknowledgements
Competing interests	26	Declare any competing interests of review authors.	N/A
Availability of data, code and other materials	27	Report which of the following are publicly available and where they can be found: template data collection forms; data extracted from included studies; data used for all analyses; analytic code; any other materials used in the review.	Data extracted found in Supplementary Materials

From: Page MJ, McKenzie JE, Bossuyt PM, Boutron I, Hoffmann TC, Mulrow CD, et al. The PRISMA 2020 statement: an updated guideline for reporting systematic reviews. BMJ 2021;372:n71. doi: 10.1136/bmj.n71

For more information, visit: http://www.prisma-statement.org/

2- Data collected

Information	Year Published	Context	Context (detailed)	Sample Number	Sample Preparation Method	Sample Demographic	Detection Method	Epitope production	Target Epitope	Control Epitope	Appropriate controls used?	Blocking Agent Used	% anti-Neu5Gc positive samples	Antibody Class Reported	IgG	IgA	IgM	Adjusted Variation (SD)	Adjusted Variation (Range)
**Akimoto S, Tahara H, Yanagawa S, Ide K, Tanaka Y, Kobayashi T, Ohdan H. Heterophile carbohydrate antigen N-glycolylneuraminic acid as a potential biomarker in patients with hepatocellular carcinoma. Cancer Rep (Hoboken). 2023;6(8):e1831.**	2023	Cancer	Neu5Gc antigen and antibodies as a hepatocellular carcinoma biomarker	100	Serum	Asia	ELISA	Synthetic	Neu5Gc-PAA	Neu5Ac-PAA	TRUE	OVA	53	IgG	0.06 (OD)	NOT STATED	NOT STATED	0.17	NOT STATED
**Alfonso S, Valdés-Zayas A, Santiesteban ER, Flores YI, Areces F, Hernández M, et al. A Randomized, Multicenter, Placebo-Controlled Clinical Trial of Racotumomab-Alum Vaccine as Switch Maintenance Therapy in Advanced Non-Small Cell Lung Cancer Patients. CLINICAL CANCER RESEARCH. 2014;20(14):3660-71.**	2014	Cancer	NSCLC patients immunized with a vaccine to mount an immune response against Neu5Gc-containing GM3	24	Serum	North America	ELISA	Natural	Neu5Gc-GM3	Uncoated wells	FALSE	BSA	95.8	IgG, IgM	1 in 6400 (dilution)	NOT STATED	NOT STATED	NOT STATED	NOT STATED

**Amon R, Ben-Arye SL, Engler L, Yu H, Lim N, Berre LL, et al. Glycan microarray reveal induced IgGs repertoire shift against a dietary carbohydrate in response to rabbit anti-human thymocyte therapy. Oncotarget. 2017;8(68):112236-44.**	2017	Biotherapeutics	Diabetes patients treated with anti-thrombocyte thymoglobulin	7	Serum	North America	Glycan Array	Synthetic	Matched Sialoglycan Pairs	Matched sialoglycan pairs	TRUE	OVA	100	IgG	30.8 ug/mL	NOT STATED	NOT STATED	0.44	NOT STATED
**Angeletti A, Bruschi M, Kajana X, Lugani F, Candiano G, Ghiggeri GM. Anti-Neu5Gc Antibodies do not Affect Response to Human or Chimeric Monoclonal Anti-CD20 Antibodies in Children with Nephrotic Syndrome. J Am Soc Nephrol. 2022;33(11):1985-7.**	2022	Inflammation	Anti-Neu5Gc in children with Nephrotic syndrome as pre-screening for a trial of anti-CD20 mAbs	101	Serum	Europe	ELISA	Natural	Bovine Sialomucin	NOT STATED	FALSE	OVA	NOT STATED	IgG	0.9 (OD)	NOT STATED	NOT STATED	0.211	NOT STATED
**Apostolovic D, Tran TA, Sánchez-Vidaurre S, Cirkovic Velickovic T, Starkhammar M, Hamsten C, van Hage M. Red meat allergic patients have a selective IgE response to the α-Gal glycan. Allergy. 2015;70(11):1497-500.**	2015	Inflammation	Anti-Neu5Gc IgE in patients with red meat allergies	24	IgE only	Europe	ImmunoCAP	Synthetic	Neu5Gc-Biotin	NOT STATED	FALSE	NOT STATED	0	IgE	NOT STATED	NOT STATED	NOT STATED	NOT STATED	NOT STATED

**Arita K, Ikuta K, Nishi Y, Kato S, Yamauchi E, Maki S, naiki M. Heterophile Hanganutziu-Deicher antibodies in sera of patients with Kawasaki diseases. Biken J. 1982;25(4):157-62.**	1982	Inflammation	HD antibodies in patients with Kawasaki disease	39	Serum	Asia	ELISA	Natural	Neu5Gc-GM3	Uncoated wells	FALSE	NONE	35.1	IgG	1.88 (RIA)	NOT STATED	NOT STATED	0.55	NOT STATED
**Bashir S, Ben Arye SL, Reuven EM, Yu H, Costa C, Galiñanes M, et al. Presentation Mode of Glycans Affect Recognition of Human Serum anti-Neu5Gc IgG Antibodies. BIOCONJUGATE CHEMISTRY. 2019;30(1):161-8.**	2019	Healthy Population	Testing variation in anti-Neu5Gc titres associated with different ways of presenting Neu5Gc	5	Serum	Middle East	ELISA	Natural	WT Mouse Sialoglycoproteins	CMAH KO mouse Sialoglycoproteins	TRUE	OVA	100	IgG	0.69 (OD)	NOT STATED	NOT STATED	0.855	NOT STATED
**Bashir S, Ben Arye SL, Reuven EM, Yu H, Costa C, Galiñanes M, et al. Presentation Mode of Glycans Affect Recognition of Human Serum anti-Neu5Gc IgG Antibodies. BIOCONJUGATE CHEMISTRY. 2019;30(1):161-8.**	2019	Healthy Population	Variation in anti-Neu5Gc titres associated with different ways of presenting Neu5Gc	11	Serum	Middle East	Glycan Array	Synthetic	Matched Sialoglycan Pairs	Matched sialoglycan pairs	TRUE	OVA	100	IgG	2379 (RFU)	NOT STATED	NOT STATED	0.988	NOT STATED

**Bashir S, Fezeu LK, Leviatan Ben-Arye S, Yehuda S, Reuven EM, Szabo de Edelenyi F, et al. Association between Neu5Gc carbohydrate and serum antibodies against it provides the molecular link to cancer: French NutriNet-Santé study. BMC Med. 2020;18(1):262.**	2020	Healthy Population	Testing links between diet and anti-Neu5Gc antibodies using a large scale nutritional survey	120	Serum	Europe	ELISA	Natural	WT Mouse Sialoglycoproteins	CMAH KO mouse Sialoglycoproteins	TRUE	OVA	100	IgG	4.4 (ug/mL)	NOT STATED	NOT STATED	0.886	NOT STATED
**Bashir S, Fezeu LK, Leviatan Ben-Arye S, Yehuda S, Reuven EM, Szabo de Edelenyi F, et al. Association between Neu5Gc carbohydrate and serum antibodies against it provides the molecular link to cancer: French NutriNet-Santé study. BMC Med. 2020;18(1):262.**	2020	Healthy Population	Testing links between diet and anti-Neu5Gc antibodies using a large scale nutritional survey	120	Serum	Europe	Glycan Array	Synthetic	Matched Sialoglycan Pairs	Matched sialoglycan pairs	TRUE	OVA	100	IgG	NOT STATED	NOT STATED	NOT STATED	NOT STATED	NOT STATED
**Basnet NB, Ide K, Tahara H, Tanaka Y, Ohdan H. Deficiency of N-glycolylneuraminic acid and Galα1-3Galβ1-4Glcnac epitopes in xenogeneic cells attenuates cytotoxicity of human natural antibodies. Xenotransplantation. 2010;17(6):440-8.**	2010	Healthy population	Assessing cytotoxicity of anti-Neu5Gc antibodies towards thymocytes from WT and CMAH KO mice	7	Serum, heat inactivated	Asia	Flow Cytometry	Natural	Mouse Thymocytes	CMAH KO mouse thymocytes	TRUE	NONE	NOT STATED	IgG, IgM	25 (MFI)	NOT STATED	8 (MFI)	NOT STATED	0.88

**Ben-Arye SL, Schneider C, Yu H, Bashir S, Chen X, von Gunten S, Padler-Karavani V. Differential Recognition of Diet-Derived Neu5Gc-Neoantigens on Glycan Microarrays by Carbohydrate-Specific Pooled Human IgG and IgA Antibodies. BIOCONJUGATE CHEMISTRY. 2019;30(5):1565-74.**	2019	Healthy population	Assessing capability of different microarray preparations to detect anti-Neu5Gc antibodies in serum	Pooled	IVIG, 7 different preparations	NA	Glycan Array	Synthetic	Matched Sialoglycan Pairs	Matched sialoglycan pairs	TRUE	OVA	100	IgG, IgA	950 (RFU)	1100 (RFU)	NOT STATED	NOT STATED	10.78
**Ben-Arye SL, Yu H, Chen X, Padler-Karavani V. Profiling Anti-Neu5Gc IgG in Human Sera with a Sialoglycan Microarray Assay. JOVE-JOURnaL OF VISUALIZED EXPERIMENTS. 2017(125).**	2017	Healthy Population	Protocol for using glycan arrays to assess anti-Neu5Gc antibodies in human serum	12	Serum	Middle East	Glycan Array	Synthetic	Matched Sialoglycan Pairs	Matched sialoglycan pairs	TRUE	OVA	100	IgG	2.1 (ug/mL)	NOT STATED	NOT STATED	NOT STATED	18.05
**Blixt O, Kumagai-Braesch M, Tibell A, Groth CG, Holgersson J. Anticarbohydrate antibody repertoires in patients transplanted with fetal pig islets revealed by glycan arrays. American Journal of Transplantation. 2009;9(1):83-90.**	2009	Xenotransplantation	Anti-Neu5Gc antibody titres in type 1 Diabetes patients transplanted with porcine fetal islet cells	7	Serum, stored at -70C	Europe	Glycan Array	Synthetic	Neu5Gc-containing glycans	Neu5Ac-containing glycans	TRUE	NOT STATED	28	Total	15000 (RFU)	NOT STATED	NOT STATED	NOT STATED	2.33

**Boligan KF, Oechtering J, Keller CW, Peschke B, Rieben R, Bovin N, et al. Xenogeneic Neu5Gc and self-glycan Neu5Ac epitopes are potential immune targets in MS. Neurol Neuroimmunol Neuroinflamm. 2020;7(2).**	2020	Inflammation	Anti-Neu5Gc antibodies in patients with relapsing-remitting multiple sclerosis.	25	Serum, stored at -80C	Europe	Glycan Array	Synthetic	a2-3 Neu5Gc	a2-3 Neu5Ac	TRUE	BSA	96	IgG	215 (RFU)	NOT STATED	NOT STATED	NOT STATED	4.65
**Burlak C, Paris LL, Lutz AJ, Sidner RA, Estrada J, Li P, et al. Reduced binding of human antibodies to cells from GGTA1/CMAH KO pigs. Am J Transplant. 2014;14(8):1895-900.**	2014	Xenotransplantation	Binding of anti-Neu5Gc antibodies to GGTA1/CMAH KO pig cells	121	Serum, heat inactivated	North America	Flow Cytometry	Natural	Pig PBMCs	GGTA1/CMAH KO pig PBMCs	TRUE	NOT STATED	96.6	IgG, IgM	1000 (MFI)	NOT STATED	1500 (MFI)	NOT STATED	4.1
**Butler JR, Martens GR, Estrada JL, Reyes LM, Ladowski JM, Galli C, et al. Silencing porcine genes significantly reduces human-anti-pig cytotoxicity profiles: an alternative to direct complement regulation. TRANSGENIC RESEARCH. 2016;25(5):751-9.**	2016	Xenotransplantation	Binding of anti-Neu5Gc antibodies to GGTA1/CMAH/B4GalNT2 KO pig cells	10	Serum, heat inactivated	North America	Flow Cytometry	Natural	Pig PBMCs	CMAH KO PBMCs	TRUE	NOT STATED	NOT STATED	IgG, IgM	3917.1 (MFI)	NOT STATED	5615.2 (MFI)	0.27	NOT STATED

**Caldwell KE, Cayer M, Whitney PL, Fletcher MA. Immunochemical studies of infectious mononucleosis-X. Characterization of a glycoprotein from horse erythrocytes which reacts with Paul-Bunnell antibody. Molecular Immunology. 1982;19(6):779-91.**	1982	Infection	Binding of human antibodies to Neu5Gc-containing glycoproteins (isolated from horse erythrocytes) in infectious mononucleosis	NOT STATED	Serum, heterophile positive	North America	Radioimmunoassay	Natural	Horse erythrocyte glycoprotein	NOT STATED	FALSE	Gelatin	NOT STATED	NOT STATED	5 (uM)	NOT STATED	NOT STATED	NOT STATED	NOT STATED
**Carpo M, Nobile-Orazio E, Chigorno V, Sonnino S. The anti-oligosaccharide antibodies present in sera from patients with motor neuron disease and neuropathy recognize the N-glycolylneuraminic acid containing gangliotetrahexosyl oligosaccharide. Glycoconj J. 1995;12(5):729-31.**	1995	Inflammation	Serum antibodies against both Neu5Gc-GM1 and Neu5Ac-GM1 in patients with motor neuron disease	2	Serum	Europe	TLC-immunostaining	Natural	Neu5Gc-GM1	Neu5Ac-GM1	TRUE	NOT STATED	100	IgM	NOT STATED	NOT STATED	NOT STATED	NOT STATED	NOT STATED

**Chaban R, Habibabady Z, Hassanein W, Connolly MR, Burdorf L, Redding E, et al. Knock-out of N-glycolylneuraminic acid attenuates antibody-mediated rejection in xenogenically perfused porcine lungs. Xenotransplantation. 2022;29(6):e12784.**	2022	Xenotransplantation	Binding of human serum antibodies to lung tissue from GalT/CMAH KO pigs. Measured titres pre- and post-incubation with porcine lung tissue	2	Plasma, collected in EDTA	North America	Flow Cytometry	Natural	porcine CMAH HEK cells	WT HEK cells	TRUE	BSA	100	IgM, IgG	66	NOT STATED	55	0.364	NOT STATED
**Chihara RK, Lutz AJ, Paris LL, Wang ZY, Sidner RA, Heyrman AT, et al. Fibronectin from alpha 1,3-galactosyltransferase knockout pigs is a xenoantigen. J Surg Res. 2013;184(2):1123-33.**	2013	Xenotransplantation	Antibody binding to cells from GTKO pigs, with a specific focus on Neu5Gc conjugates on fibronectin	1	Serum, heat inactivated	NOT STATED	ELISA	Synthetic	Neu5Gc-PAA	Neu5Ac-PAA	TRUE	HSA	100	IgG	0.008	NOT STATED	NOT STATED	NOT STATED	NOT STATED
**Couvrat-Desvergnes G, Salama A, Le Berre L, Evanno G, Viklicky O, Hruba P, et al. Rabbit antithymocyte globulin-induced serum sickness disease and human kidney graft survival. J Clin Invest. 2015;125(12):4655-65.**	2015	Biotherapeutics	Anti-Neu5Gc antibodies induced by treatment of transplant patients with rabbit ATG as a hypothesised driver of serum sickness and kidney graft rejection	889	Serum	Europe	ELISA	Natural	WT Mouse Sialoglycoproteins	WT Mouse sialoglycoproteins + pre-incubation with 1:4000 CMAH KO mouse sialoglycoproteins	FALSE	OVA	73.1	IgG	2.28	NOT STATED	NOT STATED	1.35	NOT STATED

**Cross-najafi AA, Farag K, Isidan A, Li W, Zhang W, Lin Z, et al. Co-expression of HLA-E and HLA-G on genetically modified porcine endothelial cells attenuates human NK cell-mediated degranulation. Front Immunol. 2023;14:1217809.**	2023	Xenotransplantation	Human NK cell responses and antibody production against porcine liver endothelial cells with KO of CMAH and 4 other 'xenoantigen-associated' genes	20	Serum, heat inactivated	North America	Flow Cytometry	Natural	CMAH/GGTA1/B4GalNT2/SLA-Ia chain/B2-microglobulin KO Porcine liver endothelial cells	NOT STATED	FALSE	NOT STATED	NOT STATED	IgG, IgM	3400 (MFI)	NOT STATED	2600 (MFI)	0.26	NOT STATED
**Díaz A, Alfonso M, Alonso R, Saurez G, Troche M, Catalá M, et al. Immune responses in breast cancer patients immunized with an anti-idiotype antibody mimicking NeuGc-containing gangliosides. CLINICAL IMMUNOLOGY. 2003;107(2):80-9.**	2003	Cancer	Anti-Neu5Gc-ganglioside antibodies following immunization of breast cancer patients with a monoclonal antibody Neu5Gc-GM3 mimetic (1E10)	10	serum	North America	ELISA	Natural	Neu5Gc-GM3	Neu5Gc-GM3 + pre-incubation with 1E10	FALSE	BSA	100	IgG, IgM	1 in 1840	NOT STATED	1 in 5640	NOT STATED	3.26
**Diswall M, Angström J, Karlsson H, Phelps CJ, Ayares D, Teneberg S, Breimer ME. Structural characterization of alpha1,3-galactosyltransferase knockout pig heart and kidney glycolipids and their reactivity with human and baboon antibodies. Xenotransplantation. 2010;17(1):48-60.**	2010	Xenotransplantation	Reactivity of human serum antibodies to GalT-KO pig heart and kidney tissue. Identification of Neu5Gc as a 'non-gal' xenoantigen	21	Serum, pooled, AB blood group	Europe	Thin layer chromatography immunostaining	Natural	Glycolipid fractions from porcine tissue	Glycolipid fractions from GALT KO porcine tissue	FALSE	NOT STATED	NOT STATED	IgG, IGM, IgA	NOT STATED	NOT STATED	NOT STATED	NOT STATED	NOT STATED

**Eckermann JM, Buhler LH, Zhu A, Dor FJMF, Awwad M, Cooper DKC. Initial investigation of the potential of modified porcine erythrocytes for transfusion in primates. Xenotransplantation. 2004;11(1):18-26.**	2004	Xenotransplantation	Binding of 'non-Gal' antibodies in human serum to galactosidase treated porcine RBCs	NOT STATED	Serum, heat inactivated	Europe	Flow Cytometry	Natural	porcine RBCs	Galactosidase treated porcine RBCs	FALSE	BSA	NOT STATED	IgM	NOT STATED	NOT STATED	11 (MFI)	NOT STATED	NOT STATED
**Eleftheriou P, Kynigopoulos S, Giovou A, Mazmanidi A, Yovos J, Skepastianos P, et al. Prevalence of anti-Neu5Gc antibodies in patients with hypothyroidism. Biomed Res Int. 2014;2014:963230.**	2014	Inflammation	Anti-Neu5Gc antibodies in patients with hypothyroidism	48	Serum. Collected via centrifugation at 2500xg after 30 min clotting. Stored at -20C	NOT STATED	ELISA	Synthetic	Neu5Gc monomer	Neu5Ac monomer	TRUE	OVA	98	IgG	14.8	NOT STATED	NOT STATED	1	NOT STATED
**Estrada JL, Martens G, Li P, Adams A, Newell KA, Ford ML, et al. Evaluation of human and non-human primate antibody binding to pig cells lacking GGTA1/CMAH/4GalNT2 genes. XENOTRANSPLANTATION. 2015;22(3):194-202.**	2015	Xenotransplantation	Human serum antibody binding to GGTA1/CMAH/B4GalNT2 KO porcine PBMCs	82	Serum, heat inactivated	North America	Flow Cytometry	Natural	porcine PBMCs	CMAH KO porcine PBMCs	TRUE	NOT STATED	NOT STATED	IgG, IgM	NOT STATED	NOT STATED	NOT STATED	NOT STATED	NOT STATED

**Feng H, Li T, Du J, Xia Q, Wang L, Chen S, et al. Both natural and Induced Anti-Sda Antibodies Play Important Roles in GTKO Pig-to-Rhesus Monkey Xenotransplantation. Front Immunol. 2022;13:849711.**	2022	Xenotransplantation	Human serum antibody binding to CMAH KO porcine PBMCs	20	Serum, stored at -80, heat inactivated immediately before use	Asia	Flow Cytometry	Natural	porcine PBMCs	CMAH KO porcine PBMCs	TRUE	Goat Serum	100	IgG, IgM	1000 (MFI)	NOT STATED	1700 (MFI)	NOT STATED	0.1
**Fischer K, Rieblinger B, Hein R, Sfriso R, Zuber J, Fischer A, et al. Viable pigs after simultaneous inactivation of porcine MHC class I and three xenoreactive antigen genes GGTA1, CMAH and B4GALNT2. Xenotransplantation. 2020;27(1):e12560**	2020	Xenotransplantation	Human serum antibody binding to CMAH KO porcine kidney cells	NOT STATED	Serum, collected in S-monovette tubes. 30 min clotting then centrifugation at 2000xg. Stored at -80C	Europe	Immunofluorescence staining	Natural	porcine kidney fibroblasts	CMAH KO porcine kidney fibroblasts	TRUE	BSA	100	IgG, IgM	52000000 (int. den.)	NOT STATED	37000000 (int. den.)	0.538	NOT STATED
**Frei R, Ferstl R, Roduit C, Ziegler M, Schiavi E, Barcik W, et al. Exposure to nonmicrobial N-glycolylneuraminic acid protects farmers' children against airway inflammation and colitis. J Allergy Clin Immunol. 2018;141(1):382-90.e7.**	2018	Inflammation	Anti-Neu5Gc antibodies in children from rural environments, with a focus on links to inflammatory disease	299	Serum	Europe	ELISA	Synthetic	Neu5Gc-PAA	Neu5Ac-PAA	TRUE	TBST	NOT STATED	IgG	2	NOT STATED	NOT STATED	2.935	NOT STATED

**Frei R, Roduit C, Ferstl R, O'Mahony L, Lauener RP. Exposure of Children to Rural Lifestyle Factors Associated With Protection Against Allergies Induces an Anti-Neu5Gc Antibody Response. Front Immunol. 2019;10:1628.**	2019	Inflammation	Anti-Neu5Gc antibodies in children from rural environments, with a focus on links to allergic disease. Compared children with cows milk vs no milk consumption	734	Serum	Europe	ELISA	Synthetic	Neu5Gc-PAA	Neu5Ac-PAA	TRUE	TBST	NOT STATED	IgG	5.57 (ratio to no cows milk)	NOT STATED	NOT STATED	3.056	NOT STATED
**Gao B, Long C, Lee W, Zhang Z, Gao X, Landsittel D, et al. Anti-Neu5Gc and anti-non-Neu5Gc antibodies in healthy humans. PLoS One. 2017;12(7):e0180768.**	2017	Healthy Population	Assessing whether anti-Neu5Gc antibodies are present in healthy human serum via ability to bind to WT and CMAH KO porcine RBCs and AECs	84	Serum, heat inactivated, stored at -80C	Global	Flow Cytometry	Natural	porcine RBCs and porcine AECs	CMAH KO porcine RBCs and porcine AECs	TRUE	Goat Serum	50	IgG, IgM	25 (relative GM)	NOT STATED	30 (relative GM)	NOT STATED	6
**Gao B, Long C, Lee W, Zhang Z, Gao X, Landsittel D, et al. Anti-Neu5Gc and anti-non-Neu5Gc antibodies in healthy humans. PLoS One. 2017;12(7):e0180768.**	2017	Healthy Population	Assessing whether anti-Neu5Gc antibodies are present in healthy human serum via ability to bind to WT and CMAH KO porcine RBCs and AECs	84	Serum, heat inactivated, stored at -80C	Global	ELISA	Synthetic	Neu5Gc-PAA	Neu5Ac-2-OCH2C6H4-p-NHCOCH2	FALSE	BSA	71	IgG, IgM	0.15	NOT STATED	0.02	NOT STATED	5
**Gathuru JK, Higashi H, Kato S, Usuba O, naiki M. Use of biotinylated antibody for the assay of Hanganutziu-Deicher antibodies and antigens in fluids and tissues from cancer patients. Jpn J Vet Res. 1989;37(2):71-83.**	1989	Cancer	Attempt to improve ELISA assays to detect anti-Neu5Gc-GM3 (HD antibody) concentration in human samples. Inferred anti-Neu5Gc in human serum via the ability of sera to outcompete a biotinylated chicken anti-Neu5Gc-GM3 antibody	3	Serum	Asia	ELISA	Natural	Neu5Gc-GM3	Neu5Ac-GM3	TRUE	OVA	66	IgG, IgM, IgA	50% inhibition	NOT STATED	NOT STATED	0.2	NOT STATED

**Ghaderi D, Taylor RE, Padler-Karavani V, Diaz S, Varki A. Implications of the presence of N-glycolylneuraminic acid in recombinant therapeutic glycoproteins. nat Biotechnol. 2010;28(8):863-7.**	2010	Biotherapeutics	Assessed binding of affinity-purified anti-Neu5Gc antibodies from human serum to Cetuximab and Panitumumab	NOT STATED	Serum	North America	ELISA	Natural	Cetuximab	Sialidase-treated cetuximab	FALSE	TBST	NOT STATED	IgG	0.21 (OD)	NOT STATED	NOT STATED	0.476	NOT STATED
**Gołaszewska E, Kurowska E, Duk M, Kościelak J. Paul-Bunnell antigen and a possible mechanism of formation of heterophile antibodies in patients with infectious mononucleosis. Acta Biochim Pol. 2003;50(4):1205-11.**	2003	Infection	Neu5Gc-containing glycolipids as a component of the 'Paul-Bunnel' antigen used to test for Infectious Mononucleosis	3	Serum, pooled	Europe	ELISA	Natural	Neu5Gc-GM3 (horse)	Neu5Ac-GM3 (dog)	TRUE	PBST	NOT STATED	IgM	NOT STATED	NOT STATED	75% inhibition	NOT STATED	NOT STATED
**He S, Li T, Feng H, Du J, Cooper DKC, Hara H, et al. Incidence of serum antibodies to xenoantigens on triple-knockout pig cells in different human groups. Xenotransplantation. 2023:e12818.**	2023	Xenotransplantation	Binding of human serum antibodies from healthy patients, renal disease patients and kidney transplant patients to PBMCs from triple KO pigs	130	Serum, stored at -80C	Asia	Flow Cytometry	Natural	porcine PBMCs	NOT STATED	FALSE	NOT STATED	86 (IgM), 0 (IgG)	IgG, IgM	1 (GM)	NOT STATED	2.1 (GM)	NOT STATED	4.1

**Hernández AM, Rodríguez N, González JE, Reyes E, Rondón T, Griñán T, et al. Anti-NeuGcGM3 Antibodies, Actively Elicited by Idiotypic Vaccination in Nonsmall Cell Lung Cancer Patients, Induce Tumor Cell Death by an Oncosis-Like Mechanism. JOURnaL OF IMMUNOLOGY. 2011;186(6):3735-44.**	2011	Cancer	Attempts to elicit anti-Neu5Gc-GM3 antibodies in patients to target NSCLC cells. Measured pre- and post-immunized sera binding to Neu5Gc-GM3-expressing L1210 murine lymphocytic leukaemia cells	4	Serum	North America	Flow Cytometry	Natural	L1210 cells	NOT STATED	FALSE	BSA	66	IgG	58.68% (avg. shift)	NOT STATED	NOT STATED	NOT STATED	0.9
**Hernández AM, Toledo D, Martínez D, Griñán T, Brito V, Macías A, et al. Characterization of the Antibody Response against NeuGcGM3 Ganglioside Elicited in Non-Small Cell Lung Cancer Patients Immunized with an Anti-Idiotype Antibody. JOURnaL OF IMMUNOLOGY. 2008;181(9):6625-34.**	2008	Cancer	Attempts to elicit anti-Neu5Gc-GM3 antibodies in patients to target NSCLC cells. Measured pre- and post-immunized sera binding to Neu5Gc-GM3-expressing X63 murine myeloma cells	20	Serum	North America	Flow Cytometry	Natural	X63 murine lung cancer cells	H82 human lung cancer cells	FALSE	NOT STATED	83	IgG, IgM	45.5% (avg. shift)	NOT STATED	NOT STATED	NOT STATED	0.627

**Hernández AM, Toledo D, Martínez D, Griñán T, Brito V, Macías A, et al. Characterization of the Antibody Response against NeuGcGM3 Ganglioside Elicited in Non-Small Cell Lung Cancer Patients Immunized with an Anti-Idiotype Antibody. JOURnaL OF IMMUNOLOGY. 2008;181(9):6625-34.**	2008	Cancer	Attempts to elicit anti-Neu5Gc-GM3 antibodies in patients to target NSCLC cells. Measured pre- and post-immunized sera binding to Neu5Gc-GM3-expressing X63 murine myeloma cells	20	Serum	North America	ELISA	Natural	Neu5Gc-GM3	Uncoated wells	FALSE	BSA	80	IgG, IgM	2.9 (OD)	NOT STATED	NOT STATED	NOT STATED	0.24
**Hurh S, Kang B, Choi I, Cho B, Lee EM, Kim H, et al. Human antibody reactivity against xenogeneic N-glycolylneuraminic acid and galactose-α-1,3-galactose antigen. Xenotransplantation. 2016;23(4):279-92.**	2016	Xenotransplantation	Human serum antibody binding and CDC against CMAH-transfected HEK cells to invesitgate role in graft rejection	100	Serum, collected in BD Vacutainer SST tubes, stored at -80C, heat inactivated	Asia	Flow Cytometry	Natural	pCMAH HEK cells	WT HEK cells	TRUE	Neu5Gc assay blocking solution (BioLegend)	4 (IgM), 62 (IgG)	IgG, IgM	4.88 (log MFI)	NOT STATED	3.55 (log MFI)	0.299	NOT STATED
**Hurh S, Kang B, Choi I, Cho B, Lee EM, Kim H, et al. Human antibody reactivity against xenogeneic N-glycolylneuraminic acid and galactose-α-1,3-galactose antigen. Xenotransplantation. 2016;23(4):279-92.**	2016	Xenotransplantation	Human serum antibody binding and CDC against CMAH-transfected HEK cells to invesitgate role in graft rejection	20	Serum, collected in BD Vacutainer SST tubes, stored at -80C, heat inactivated	Asia	ELISA	Synthetic	Neu5Gc-PAA	PAA only	FALSE	TBST	40 (IgM), 85 (IgG)	IgG, IgM	0.09 (OD)	NOT STATED	0.09 (OD)	0.556	NOT STATED

**Kanduma EG, Mukuria JC, Mwanda OW. Serum total sialic acid and Hanganutziu-Deicher antibody in normals and in cancer patients. East Afr Med J. 2007;84(5):207-14.**	2007	Cancer	Level of 'HD antibody' in patients with various cancers	420	Serum, stored at -20C	Africa	ELISA	Natural	Horse erythrocyte membrane	sodium taurodeoxycholate	FALSE	OVA	NOT STATED	IgG	0.014 (OD)	NOT STATED	NOT STATED	3.57	NOT STATED
**Kim GA, Lee EM, Jin JX, Lee S, Taweechaipaisankul A, Hwang JI, et al. Generation of CMAHKO/GTKO/shTNFRI-Fc/HO-1 quadruple gene modified pigs. Transgenic Res. 2017;26(4):435-45.**	2017	Xenotransplantation	Binding of human serum antibodies to CMAH/GT/shTNFRI-Fc/Hhaho-1 KO pig aortic endothelial cells	Pooled	Serum, heat inactivated	North America	Flow Cytometry	Natural	porcine AECs	GT/CMAH KO porcine AECs	FALSE	NOT STATED	NOT STATED	IgG, IgM	75.48 (MFI)	NOT STATED	301.52 (MFI)	1.57	NOT STATED
**Kobayashi T, Suzuki A, Yokoyama I, Abe M, Hayashi S, nagasaka T, et al., editors. Immunogenicity of Hanganutziu-Deicher antigens in pig-to-human xenotransplantation. Transplantation Proceedings; 2000.**	2000	Xenotransplantation	Binding of human serum antibodies from patients treated with ex vivo pig kidney perfusion or fetal porcine islet transplant to 'HD-antigens' on porcine AECs	32	Serum	Asia	ELISA	Natural	Neu5Gc-GM3	Uncoated wells	FALSE	OVA	NOT STATED	IgG, IgM	0.099 (OD)	NOT STATED	0.054 (OD)	0.869	NOT STATED

**Kobayashi T, Yokoyama I, Suzuki A, Abe M, Hayashi S, Matsuda H, et al. Lack of antibody production against Hanganutziu-Deicher (H-D) antigens with N-glycolylneuraminic acid in patients with porcine exposure history. Xenotransplantation. 2000;7(3):177-80.**	2000	Xenotransplantation	Antibodies against HD antigens in humans exposed to porcine tissues for therapeutic purposes	12	Serum	Europe	ELISA	Natural	Neu5Gc-GM3	Uncoated wells	FALSE	OVA	0	IgG, IgM	0.099 (OD)	NOT STATED	0.054 (OD)	0.869	NOT STATED
**Koda T, Aosasa M, Asaoka H, nakaba H, Matsuda H. Application of tyramide signal amplification for detection of N-glycolylneuraminic acid in human hepatocellular carcinoma. Int J Clin Oncol. 2003;8(5):317-21.**	2003	Cancer	Assessing a tyramide signal amplification method for detecting low-level Neu5Gc in hepatocellular carcinoma tissue samples. Assessed anti-Neu5Gc antibodies in HCC patient sera as an aside to this.	17	Serum, separated by centrifugation, stored at -30C	Asia	ELISA	Natural	Neu5Gc-GM3 (horse erythrocytes)	NOT STATED	FALSE	OVA	64.7 (IgG), 52.9 (IgM)	IgG, IgM	0.63 (OD)	NOT STATED	0.191 (OD)	0.254	NOT STATED
**Komoda H, Miyagawa S, Kubo T, Kitano E, Kitamura H, Omori T, et al. A study of the xenoantigenicity of adult pig islets cells. XENOTRANSPLANTATION. 2004;11(3):237-46.**	2004	Xenotransplantation	Assessed binding of 'non-Gal' antibodies in human serum to porcine islet cells. Used HD antigen expression on islet cells to infer anti-Neu5Gc as accounting for a proportion of non-gal antibodies	Pooled	Serum, pooled blood type O	NOT STATED	Flow Cytometry	Natural	Porcine islet cells	PDMP (glycosylation inhibitor) treated porcine islet cells	FALSE	PBS	NOT STATED	IgG, IgM	3.05 (MFI)	NOT STATED	0.21 (OD)	NOT STATED	NOT STATED

**Kumar G, Satyananda V, Fang J, Zhou H, Fujita M, Ekser B, et al. Is There a Correlation Between Anti-Pig Antibody Levels in Humans and Geographic Location During Childhood? TRANSPLANTATION. 2013;96(4):387-93.**	2014	Xenotransplantation	Assessed anti-Neu5Gc as 'anti-non-Gal' antibodies. Donor serum from multiple geographic locations	75	Serum, heat inactivated, stored at -80C	Global	Flow Cytometry	Natural	WT porcine PBMCs	GTKO porcine PBMCs	FALSE	NOT STATED	NOT STATED	IgG, IgM	1.38 (rMFI)	NOT STATED	1.60 (rMFI)	0.11	NOT STATED
**Le Berre L, Rousse J, Gourraud PA, Imbert-Marcille BM, Salama A, Evanno G, et al. Decrease of blood anti-α1,3 Galactose Abs levels in multiple sclerosis (MS) and clinically isolated syndrome (CIS) patients. Clin Immunol. 2017;180:128-35.**	2017	Inflammation	Anti-Neu5Gc antibody titres in patients with MS (theorized due to a potential link between EBV infection and anti-Neu5Gc antibodies)	193	Serum	Europe	ELISA	Natural	WT Mouse Sialoglycoproteins	WT Mouse sialoglycoproteins + pre-incubation with 1:4000 CMAH KO mouse sialoglycoproteins	FALSE	OVA	100	IgG	2.9	NOT STATED	NOT STATED	0.172	NOT STATED
**Lee EJ, Lee H, Park EM, Kang HJ, Kim SJ, Park CG. Immunoglobulin M and Immunoglobulin G Subclass Distribution of Anti-galactose-Alpha-1,3-Galactose and Anti-N-Glycolylneuraminic Acid Antibodies in Healthy Korean Adults. Transplant Proc. 2021;53(5):1762-70.**	2021	Healthy Population	Anti-Neu5Gc antibody titres in healthy Korean adults	380	Serum	Asia	ELISA	Synthetic	Neu5Gc-BSA	Uncoated wells	FALSE	BSA	96.6 (IgG), 87.4 (IgM)	IgG, IgM	0.194	NOT STATED	0.328	NOT STATED	NOT STATED

**Lee W, Hara H, Ezzelarab MB, Iwase H, Bottino R, Long C, et al. Initial in vitro studies on tissues and cells from GTKO/CD46/NeuGcKO pigs. Xenotransplantation. 2016;23(2):137-50.**	2016	Xenotransplantation	Human serum antibody binding to tissues from GGTA/CMAH KO pigs	6	Serum, all blood groups	North America	Flow Cytometry	Natural	GGTA KO porcine aortic and corneal endothelial cells	GT/CMAH KO porcine aortic and corneal endothelial cells	TRUE	Goat Serum	NOT STATED	IgG, IgM	2 (AEC, rMFI change)	NOT STATED	3 (AEC, rMFI change)	0.25	NOT STATED
**Lee W, Long C, Ramsoondar J, Ayares D, Cooper DK, Manji RA, Hara H. Human antibody recognition of xenogeneic antigens (NeuGc and Gal) on porcine heart valves: could genetically modified pig heart valves reduce structural valve deterioration? Xenotransplantation. 2016;23(5):370-80.**	2016	Xenotransplantation	Human serum antibody binding to heart valve tissue from GTKO/CMAH KO pigs	Pooled	Serum, pooled, heat inactivated	North America	Immunofluorescence staining	Natural	GTKO pig heart valves	GT/CMAH KO pig heart valves	TRUE	Goat Serum	NOT STATED	IgG, IgM	Decrease	NOT STATED	Decrease	NOT STATED	NOT STATED
**Lee W, Miyagawa Y, Long C, Ekser B, Walters E, Ramsoondar J, et al. Expression of NeuGc on Pig Corneas and Its Potential Significance in Pig Corneal Xenotransplantation. Cornea. 2016;35(1):105-13.**	2016	Xenotransplantation	Human serum antibody binding to corneal tissue from CMAH KO pigs	Pooled	Serum, pooled, heat inactivated	North America	Flow Cytometry	Natural	GTKO pig corneal endothelial cells	GT/CMAH KO pig corneal endothelial cells	TRUE	Goat Serum	50	IgG, IgM	2 (MFI decrease)	NOT STATED	3 (MFI decrease)	NOT STATED	NOT STATED

**Leviatan Ben-Arye S, Schneider C, Yu H, Bashir S, Chen X, von Gunten S, Padler-Karavani V. Differential Recognition of Diet-Derived Neu5Gc-Neoantigens on Glycan Microarrays by Carbohydrate-Specific Pooled Human IgG and IgA Antibodies. Bioconjug Chem. 2019;30(5):1565-74.**	2019	Healthy Population	Pooled therapeutic IVIG binding to sialoglycan arrays	Pooled	IVIG, 5 different preparations	NOT STATED	Glycan Array	Synthetic	Matched Sialoglycan Pairs	Matched sialoglycan pairs	TRUE	OVA	100	IgG, IgA	1570 (RFU)	1100 (RFU)	NOT STATED	NOT STATED	1.33
**Leviatan Ben-Arye S, Yu H, Chen X, Padler-Karavani V. Profiling Anti-Neu5Gc IgG in Human Sera with a Sialoglycan Microarray Assay. J Vis Exp. 2017(125).**	2017	Healthy Population	Method for anti-Neu5Gc IgG detection using a sialoglycan microarray	12	Serum	Middle East	Glycan Array	Synthetic	Matched Sialoglycan Pairs	Matched sialoglycan pairs	TRUE	OVA	100	IgG	2 ng/uL	NOT STATED	NOT STATED	NOT STATED	19.3
**Li P, Walsh JR, Lopez K, Isidan A, Zhang W, Chen AM, et al. Genetic engineering of porcine endothelial cell lines for evaluation of human-to-pig xenoreactive immune responses. Sci Rep. 2021;11(1):13131.**	2021	Xenotransplantation	Binding of human serum antibodies to GGTA1/CMAH/ β4galNT2/SLA-I α chain/B2M KO porcine liver-derived cells	20	Serum	NOT STATED	Flow Cytometry	Natural	GGTA1/CMAH/B4GalNT2 KO porcine liver-derived cells	WT porcine liver-derived cells	FALSE	NOT STATED	NOT STATED	IgG, IgM	13000 (MFI decrease)	NOT STATED	14000 (MFI decrease)	NOT STATED	0.777

**Li T, Feng H, Du JX, Xia QB, Cooper DKC, Jiang HT, et al. Serum Antibody Binding and Cytotoxicity to Pig Cells in Chinese Subjects: Relevance to Clinical Renal Xenotransplantation. FRONTIERS IN IMMUNOLOGY. 2022;13.**	2022	Xenotransplantation	Binding of serum antibodies from Chinese renal failure patients to CMAH KO porcine PBMCs	20	Serum, all blood groups	Asia	Flow Cytometry	Natural	porcine PBMCs	CMAH KO porcine PBMCs	TRUE	NOT STATED	85 (IgG), 75 (IgM)	IgG, IgM	57 (rMFI decrease)	NOT STATED	134 (rMFI decrease)	NOT STATED	8.772
**Li Y, Fung J, Lin F. Local Inhibition of Complement Improves Mesenchymal Stem Cell Viability and Function After Administration. MOLECULAR THERAPY. 2016;24(9):1665-74.**	2016	Biotherapeutics	Binding of human anti-Neu5Gc antibodies to Neu5Gc incorporated onto mesenchymal stem cells. Anti-Neu5Gc response as a potential mediator of MSC death	Pooled	Serum, pooled, CompTech	North America	Flow Cytometry	Natural	MSCs fed Neu5Ac	MSCs not fed Neu5Ac	FALSE	NOT STATED	NOT STATED	IgG, IgM	Present (weak)	NOT STATED	Present (strong)	NOT STATED	NOT STATED
**Livingston PO, Ritter G, Srivastava P, Padavan M, Calves MJ, Oettgen HF, Old LJ. Characterization of IgG and IgM antibodies induced in melanoma patients by immunization with purified GM2 ganglioside. Cancer Res. 1989;49(24 Pt 1):7045-50.**	1989	Cancer	Antibodies against Neu5Gc-GM2 in melanoma patients immunized with GM2 + BCG adjuvant	24	Serum	North America	Dot Blot	Natural	Neu5Gc-GM2	Neu5Ac-GM2	TRUE	BSA + Goat Serum	62.5	IgG, IgM	2+	NOT STATED	NOT STATED	NOT STATED	1.5

**Lu Q, Padler-Karavani V, Yu H, Chen X, Wu SL, Varki A, Hancock WS. LC-MS analysis of polyclonal human anti-Neu5Gc xeno-autoantibodies immunoglobulin G Subclass and partial sequence using multistep intravenous immunoglobulin affinity purification and multienzymatic digestion. Anal Chem. 2012;84(6):2761-8.**	2012	Healthy Population	Screening IVIG preparations for anti-Neu5Gc antibodies	Pooled	IVIG, 4 different preparations	NOT STATED	Glycan Array	Synthetic	Matched Sialoglycan Pairs	Matched sialoglycan pairs	TRUE	OVA	100	IgG	1700 (RFU)	NOT STATED	NOT STATED	NOT STATED	1.882
**Lutz AJ, Li P, Estrada JL, Sidner RA, Chihara RK, Downey SM, et al. Double knockout pigs deficient in N-glycolylneuraminic acid and galactose α-1,3-galactose reduce the humoral barrier to xenotransplantation. Xenotransplantation. 2013;20(1):27-35.**	2013	Xenotransplantation	Human serum antibody binding to CMAH KO porcine PBMCs and RBCs	10	Serum, heat inactivated	North America	Flow Cytometry	Natural	PBMCs	CMAH KO PBMCs	TRUE	Sialix Blocking Agent	90 (IgM), 100 (IgG)	IgG, IgM	1592 (MFI decrease)	NOT STATED	10648 (MFI decrease)	NOT STATED	11.68
**Martin MJ, Muotri A, Gage F, Varki A. Human embryonic stem cells express an immunogenic nonhuman sialic acid. nat Med. 2005;11(2):228-32.**	2005	Biotherapeutics	Neu5Gc incorporated into Human Embryonic stem cells (HESCs) from culture medium. Ability of anti-Neu5Gc antibodies in serum to bind to HESCs	2	Serum, 1 high titre, 1 low titre	North America	Flow Cytometry	Natural	Human embryonic stem cells grown in normal media	Human embryonic stem cells grown in HS-containing media	FALSE	NOT STATED	NOT STATED	IgG	500 (MFI)	NOT STATED	NOT STATED	NOT STATED	0.1

**Martin PT, Kawanishi K, Ashbrook A, Golden B, Samraj A, Crowe KE, et al. Serum Antibodies to N-Glycolylneuraminic Acid Are Elevated in Duchenne Muscular Dystrophy and Correlate with Increased Disease Pathology in Cmah(-/-)mdx Mice. Am J Pathol. 2021;191(8):1474-86.**	2021	Inflammation	Anti-Neu5Gc antibodies in patients with Duchenne Muscular dystrophy	22	Serum	North America	Glycan Array	Synthetic	Matched Sialoglycan Pairs	Matched sialoglycan pairs	TRUE	OVA	100	IgG	6.4	NOT STATED	NOT STATED	0.0625	NOT STATED
**Milgrom F, Loza U, Czechowski D, Kasukawa R, Anthone R, Anthone S. "Heterophile" antigen in sera of human recipients of renal allografts. Proc Soc Exp Biol Med. 1996;213(1):80-4.**	1996	Inflammation	HD antibodies in patients following kidney transplantation	20	Serum	NOT STATED	Double Diffusion test	Natural	bovine erythrocytes	Bovine erythrocytes + Homogenized guinea pig kidney (to remove other 'heterophile' Abs)	FALSE	NONE	30	IgM	NOT STATED	NOT STATED	NOT STATED	NOT STATED	NOT STATED
**Miwa Y, Kobayashi T, nagasaka T, Liu D, Yu M, Yokoyama I, et al. Are N-glycolylneuraminic acid (Hanganutziu-Deicher) antigens important in pig-to-human xenotransplantation? Xenotransplantation. 2004;11(3):247-53.**	2004	Xenotransplantation	Antibodies in pooled human serum binding to porcine RBCs and AECs before and after neuraminidase treatment	Pooled	Serum, pooled	NOT STATED	Flow Cytometry	Natural	porcine RBCs and porcine AECs	Galactosidase and Neuraminidase-treated RBCs and AECs	FALSE	NOT STATED	NOT STATED	IgG, IgM	64 (MFI decrease)	NOT STATED	0 (MFI decrease)	NOT STATED	NOT STATED

**Moore TL, Dorner RW. 19S IgM Forssman-type heterophile antibodies in juvenile rheumatoid arthritis. Arthritis Rheum. 1980;23(11):1262-7.**	1980	Inflammation	HD antibodies in serum from children with juvenile rheumatoid arthritis	54	Serum, removed after 30 min clotting. Stored at -20C	North America	Hemolytic assay (measured hemoglobin release)	Natural	bovine erythrocytes	Guinea pig kidney cells	FALSE	NOT STATED	7	IgG, IgM	1 in 80	NOT STATED	1 in 1120	NOT STATED	8
**Morito T, Kano K, Milgrom F. Hanganutziu-Deicher antibodies in infectious mononucleosis and other diseases. J Immunol. 1982;129(6):2524-8.**	1982	Infection	HD antibodies in serum from infectious mononucleosis patients and patients with other conditions	41	Serum, heat inactivated, stored at -20C	Asia	ELISA	Natural	High molecular weight bovine erythrocyte glycoprotein	NOT STATED	FALSE	BSA	56.1 (IM), 22 (RA), 13.3 (cancer), 5 (healthy)	IgG, IgM (pooled)	0.552 (OD)	NOT STATED	NOT STATED	0.44	NOT STATED
**Mukuria CJ, Noguchi A, Suzuki E, naiki M. POTENTIAL USE OF SPECIFIC HUMAN AND CHICKEN ANTIBODIES FOR DETECTION OF HANGANUTZIU-DEICHER ANTIGEN(S) IN SERA OF CANCER-PATIENTS. JAPANESE JOURnaL OF MEDICAL SCIENCE & BIOLOGY. 1994;47(5-6):253-64.**	1994	Cancer	HD antibodies in serum from various cancer patients	8	Serum	Asia	Radioimmunoassay	Natural	radiolabelled bovine glycoprotein	NOT STATED	FALSE	NOT STATED	37.5	IgG	27.7% (titre)	NOT STATED	NOT STATED	NOT STATED	0.866

**Mukuria JC, naiki M, Hashimoto M, Kato S. A specific enzyme-linked immunosorbent assay (ELISA) procedure for detection of heterophile Hanganutziu and Deicher (HD) antibodies. J Immunol Methods. 1986;86(2):179-85.**	1986	Healthy Population	HD antibodies in serum from healthy individuals and patients hospitalized for a range of conditions	54	Serum, stored at -80C	Asia	ELISA	Natural	Neu5Gc-GM3	Uncoated wells	FALSE	OVA	5.7	IgG	35 (relative ELISA value)	NOT STATED	NOT STATED	NOT STATED	5.71
**Mukuria JC, naiki M, Hashimoto M, Nishiura K, Okabe M, Kato S. A potential radioimmunoassay system for detection of Hanganutziu-Deicher type heterophile antigen(s) and antibodies in tissues and fluids. J Immunol Methods. 1985;80(1):97-106.**	1985	Cancer	Developing a radioimmunoassay for HD antibodies in serum from cancer patients. Antibody binding causes radiolabelled bovine erythrocyte major protein to precipitate out of solution.	8	Serum, stored at -80C	Asia	Radioimmunoassay	Natural	radiolabelled bovine glycoprotein	NOT STATED	FALSE	OVA	37.5	IgG	27.6 (binding %)	NOT STATED	NOT STATED	NOT STATED	0.87
**Mukuria JC, naiki M, Kato S. Microstructure of the sialic acid moiety of N-glycolylneuraminyllactosylceramide and the elucidation of Hanganutziu and Deicher (HD) antigenicity. Immunology Letters. 1986;12(2-3):165-9.**	1985	Cancer	Assessing the immunogenicity of the HD antigen. Identification of Neu5Gc as the main 'immunogenic' component. Used serum from cancer patients.	4	Serum	Asia	ELISA	Natural	Neu5Gc-GM3	sialidase-treated Neu5Gc-GM3	FALSE	NOT STATED	100 (IgG), 75 (IgM)	IgG, IgM	2800 (max dilution)	NOT STATED	425 (max dilution)	NOT STATED	1.371

**Nguyen DH, Tangvoranuntakul P, Varki A. Effects of natural human antibodies against a nonhuman sialic acid that metabolically incorporates into activated and malignant immune cells. J Immunol. 2005;175(1):228-36.**	2005	Cancer	Incorporation of Neu5Gc into primary leukemia cells in culture.	21	Serum	North America	ELISA	Synthetic	Neu5Gc-PAA	Neu5Ac-PAA	TRUE	TBST	100	IgG, IGM, IgA	0.54	0.19	0.38	NOT STATED	11.59
**Nishimaki T, Kano K, Milgrom F. Hanganutziu-Deicher antigen and antibody in pathologic sera and tissues. J Immunol. 1979;122(6):2314-8.**	1979	Cancer	Using HD antibodies to also identify HD antigen in patients with various cancers and inflammatory conditions	118	Serum, heat inactivated, stored at -20C	North America	Hemolytic assay	Natural	Neu5Gc-GM3	NOT STATED	FALSE	NONE	15	Total	NOT STATED	NOT STATED	NOT STATED	NOT STATED	NOT STATED
**Nishimaki T, Kano K, Milgrom F. Studies on heterophile antibodies in rheumatoid arthritis. Arthritis Rheum. 1978;21(6):634-8.**	1978	Inflammation	Antibodies against sheep and bovine erythrocytes in Rheumatoid arthritis patients linked back to 'heterophile' HD antibodies in serum	10	Serum, heat inactivated	North America	Hemolytic assay (measured inhibition by HD antigen)	Natural	bovine erythrocytes	Human erythrocytes	FALSE	NONE	60	Total	NOT STATED	NOT STATED	NOT STATED	NOT STATED	NOT STATED
**Nishimaki T, Kano K, Milgrom F. Studies on immune complexes in rheumatoid arthritis. Arthritis Rheum. 1978;21(6):639-44.**	1978	Inflammation	Immune complexes formed in culture, triggered by HD-antigen and serum from patients with rheumatoid arthritis	15	Serum, off the clot after 30 min centrifugation. Heat inactivated.	North America	Anti-antibody inhibition test	Natural	Neu5Gc-GM3	NOT STATED	FALSE	NONE	47	IgG, IgM	NOT STATED	NOT STATED	NOT STATED	NOT STATED	NOT STATED

**Noguchi A, Mukuria CJ, Suzuki E, naiki M. Immunogenicity of N-glycolylneuraminic acid-containing carbohydrate chains of recombinant human erythropoietin expressed in Chinese hamster ovary cells. J Biochem. 1995;117(1):59-62.**	1995	Biotherapeutics	Neu5Gc in recombinant human erythropoietin produced by CHO cells. Antigenicity of rhEPO in human serum compared to Neu5Gc-GM3	1	Serum	Asia	ELISA	Natural	rhEPO and Neu5Gc-GM3	Uncoated wells	FALSE	OVA	100	IgG	10% inhibition (rhEPO)	NOT STATED	NOT STATED	NOT STATED	NOT STATED
**Noguchi A, naiki M, Mukuria CJ. Potential Use of Specific Human and Chicken Antibodies for Detection of Hanganutziu-Deicher Antigen (S) in Sera of Cancer Patients. Japanese Journal of Medical Science and Biology. 1994;47:253-64.**	1994	Cancer	Comparing chicken anti-Neu5Gc-GM3 with human anti-Neu5Gc-GM3 from cancer patients	8	Serum	Asia	Radioimmunoassay	Natural	radiolabelled bovine glycoprotein	radiolabelled bovine glycoprotein pre-absorbed	FALSE	OVA	37.5	IgG	27.6 (binding %)	NOT STATED	NOT STATED	NOT STATED	0.87
**Nowak JA, Jain NK, Stinson MW, Merrick JM. Interaction of bovine erythrocyte N-glycolylneuraminic acid-containing gangliosides and glycoproteins with a human Hanganutziu-Deicher serum. Mol Immunol. 1986;23(7):693-700.**	1986	Inflammation	Isolating HD antibodies from human serum collected from various hospital patients	1000	Serum	North America	Hemagglutination	Natural	bovine erythrocytes	NOT STATED	FALSE	NOT STATED	0.1	Total	NOT STATED	NOT STATED	NOT STATED	NOT STATED	NOT STATED

**Oaks M, Michel K, Downey FX, Thohan V. Xenoreactive antibodies and latent fibrin formation in VAD and cardiac transplant recipients can confound the detection and measurement of anti-AT1R antibodies. Am J Transplant. 2018;18(11):2763-71.**	2018	Biotherapeutics	Anti-Neu5Gc antibodies as a potential source of interference with anti-ATR1 antibody tests used to assess risk of graft rejection in transplant patients.	294	Serum	North America	ELISA	Synthetic	Biotinylated Neu5Gc	Biotinylated Neu5Ac	TRUE	PBST	100	IgG	3.48	NOT STATED	NOT STATED	NOT STATED	4.8
**Obukhova P, Tsygankova S, Chinarev A, Shilova N, Nokel A, Kosma P, Bovin N. Are there specific antibodies against Neu5Gc epitopes in the blood of healthy individuals? Glycobiology. 2020;30(6):395-406.**	2020	Healthy Population	Attempting to re-assess the prevalence of anti-Neu5Gc antibodies in the general population	20	Serum	Europe	ELISA	Synthetic	Neu5Gc-glycan-PAA	PAA only	FALSE	BSA	25	Total	13.8 (RFU %)	NOT STATED	NOT STATED	NOT STATED	0.87
**Odaka M, Yuki N, Nobile-Orazio E, Carpo M, Hirata K. Antibodies to GM1(NeuGc) in Guillain-Barré syndrome after ganglioside therapy. J Neurol Sci. 2000;175(2):96-106.**	2000	Inflammation	Anti-Neu5Gc antibodies in Guillain-Barre patients previously treated with bovine brain ganglioside injection	161	Serum	Europe	ELISA	Natural	Neu5Gc-GM1	Neu5Ac-GM1	TRUE	NOT STATED	14	IgG, IgM	30833 (dilution)	NOT STATED	875 (dilution)	NOT STATED	4.135

**Odaka M, Yuki N, Yoshino H, Kasama T, Handa S, Irie F, et al. N-glycolylneuraminic acid-containing GM1 is a new molecule for serum antibody in Guillain-Barré syndrome. Ann Neurol. 1998;43(6):829-34.**	2004	Inflammation	Anti-Neu5Gc-GM1 antibodies in Guillain-Barre syndrome patients previously injected with bovine brain gangliosides	20	Serum	Asia	ELISA	Natural	Neu5Gc-GM1	Neu5Ac-GM1	TRUE	NOT STATED	10	IgG, IgM	8000 (median dilution)	NOT STATED	4000 (median dilution)	NOT STATED	64
**Okamura RM, Lebkowski J, Au M, Priest CA, Denham J, Majumdar AS. Immunological properties of human embryonic stem cell-derived oligodendrocyte progenitor cells. J Neuroimmunol. 2007;192(1-2):134-44.**	2007	Biotherapeutics	Concerns about anti-Neu5Gc antibodies as a cause of rejection in human embryonic stem cell derived oligodendrocyte progenitor cell transplant afer spinal cord injury	4	Serum, heat inactivated	NOT STATED	ELISA	Synthetic	Neu5Gc-PAA	Neu5Ac-PAA	TRUE	TBST	75	IgG	262.5	NOT STATED	NOT STATED	NOT STATED	3.81
**Omori T, Nishida T, Komoda H, Fumimoto Y, Ito T, Sawa Y, et al. A study of the xenoantigenicity of neonatal porcine islet-like cell clusters (NPCC) and the efficiency of adenovirus-mediated DAF (CD55) expression. Xenotransplantation. 2006;13(5):455-64.**	2006	Xenotransplantation	Reactivity of human serum antibodies to HD antigens on neonatal porcine islet-like cell clusters used for transplantation in T1 diabetes patients	Pooled	Pooled Serum, blood type O	NOT STATED	Flow Cytometry	Natural	Neuraminidase-treated porcine islet-like cells	Secondary only	FALSE	NOT STATED	NOT STATED	IgG, IgM	307.7 (shift)	NOT STATED	minus 8.3 (shift)	NOT STATED	NOT STATED

**Padler-Karavani V, Hurtado-Ziola N, Pu M, Yu H, Huang S, Muthana S, et al. Human xeno-autoantibodies against a non-human sialic acid serve as novel serum biomarkers and immunotherapeutics in cancer. Cancer Res. 2011;71(9):3352-63.**	2011	Cancer	Affinity purification of anti-Neu5Gc antibodies from pooled human serum as a potential immunotherapeutic strategy. Also assessed repertoire of anti-Neu5Gc antibodies in cancer patients.	386	Serum	North America	Glycan Array	Synthetic	Matched Sialoglycan Pairs	Matched sialoglycan pairs	TRUE	OVA	NOT STATED	IgG	5000 (RFU)	NOT STATED	NOT STATED	NOT STATED	1.46
**Padler-Karavani V, Tremoulet AH, Yu H, Chen X, Burns JC, Varki A. A simple method for assessment of human anti-Neu5Gc antibodies applied to Kawasaki disease. PLoS One. 2013;8(3):e58443.**	2013	Inflammation	Assessment of anti-Neu5Gc antibodies in children with Kawasaki disease (pediatric inflammatory heart condition)	10	Serum, off the clot. Stored at -80C	North America	ELISA	Natural	WT Mouse Sialoglycoproteins	CMAH KO mouse Sialoglycoproteins	TRUE	OVA	100	IgG, IgA	2.7 (ug/mL)	0.5 (ug/mL)	NOT STATED	0.148	NOT STATED
**Padler-Karavani V, Yu H, Cao H, Chokhawala H, Karp F, Varki N, et al. Diversity in specificity, abundance, and composition of anti-Neu5Gc antibodies in normal humans: potential implications for disease. Glycobiology. 2008;18(10):818-30.**	2008	Healthy Population	Assessing the repertoire of anti-Neu5Gc antibodies in the 'normal' human population.	16	Serum, stored at -80C	North America	ELISA	Synthetic	Neu5Gc-PAA-Biotin	Neu5Ac-PAA-Biotin	TRUE	OVA	100	IgG, IgA, IgM	3.48 (ug/mL)	1.48 (ug/mL)	0.24 (ug/mL)	0.23	NOT STATED

**Pazynina GV, Tsygankova SV, Ryzhov IM, Shilova NV, Polyakova SM, Paramonov AS, et al. Synthesis of GalNGcα1-3Galnacα disaccharide and its interaction with human blood antibodies. MENDELEEV COMMUNICATIONS. 2019;29(3):254-5.**	2019	Healthy Population	Serum antibody binding to a Neu5Gc-containing version of the mucin core 5 motif (GalNGca1-3Galnaca-)	Pooled	IVIG, 1 preparation	NOT STATED	Glycan Array	Synthetic	GalNGca1-3Galnaca-sp	Galnaca1-3Galnaca-sp	TRUE	PBST	NOT STATED	IgG	3010 (AU)	NOT STATED	NOT STATED	0.095	NOT STATED
**Pham T, Gregg CJ, Karp F, Chow R, Padler-Karavani V, Cao H, et al. Evidence for a novel human-specific xeno-auto-antibody response against vascular endothelium. Blood. 2009;114(25):5225-35.**	2009	Inflammation	Potential role of anti-Neu5Gc antibodies in endothelial activation	14	Serum, stored at -80C, not heat inactivated	North America	Flow Cytometry	Natural	Human umbilical vein endothelial cells loaded with Neu5Gc	Human umbilical vein endothelial cells loaded with Neu5Ac	TRUE	NOT STATED	57	IgG, IgM	14.3 (% of standard)	NOT STATED	30.4 (% of standard)	NOT STATED	7.69
**Ramm R, Hartmann T, Tudorache I, Haverich A, Hilfiker A. No evidence for αGal epitope transfer from media containing FCS onto human endothelial cells in culture. Xenotransplantation. 2015;22(5):345-55.**	2015	Biotherapeutics	incorporation of xenogenic epitopes from FCS in media onto human endothelial cells	14	Serum, heat inactivated, stored at -80C	Europe	ELISA	Synthetic	Neu5Gc-PAA	PAA only	FALSE	Polyethylene Glycol 600	50	Total	500 (AU)	NOT STATED	NOT STATED	NOT STATED	6

**Reuven EM, Leviatan Ben-Arye S, Marshanski T, Breimer ME, Yu H, Fellah-Hebia I, et al. Characterization of immunogenic Neu5Gc in bioprosthetic heart valves. Xenotransplantation. 2016;23(5):381-92.**	2016	Xenotransplantation	Reactivity of human anti-Neu5Gc antibodies with Neu5Gc on bioprosthetic heart valves	Pooled	IVIG, 1 preparation	NOT STATED	Glycan Array	Synthetic	Matched Sialoglycan Pairs	Matched sialoglycan pairs	TRUE	OVA	NOT STATED	IgG	20779 (RFU)	NOT STATED	NOT STATED	NOT STATED	1.148
**Rodríguez-Zhurbenko N, Martínez D, Blanco R, Rondón T, Griñán T, Hernández AM. Human antibodies reactive to NeuGcGM3 ganglioside have cytotoxic antitumor properties. EUROPEAN JOURnaL OF IMMUNOLOGY. 2013;43(3):826-37.**	2013	Cancer	Assessing anti-Neu5Gc-GM3 in serum from healthy donors and NSCLC patients, as well as the ability of these antibodies to trigger CDC against murine leukaemia cells in culture	100	Serum, heat inactivated, stored at -20C	North America	ELISA	Natural	Neu5Gc-GM3	Neu5Ac-GM3	TRUE	HSA	65	IgG+M	0.1 (OD)	NOT STATED	NOT STATED	NOT STATED	4
**Rousse J, Salama A, Leviatan Ben-Arye S, Hruba P, Slatinska J, Evanno G, et al. Quantitative and qualitative changes in anti-Neu5Gc antibody response following rabbit anti-thymocyte IgG induction in kidney allograft recipients. Eur J Clin Invest. 2019;49(4):e13069.**	2019	Biotherapeutics	Anti-Neu5Gc antibodies in kidney transplant patients treated with rabbit anti-thymocyte globulin (ATG) as an immunosuppressant	60	Serum	Europe	ELISA	Natural	WT Mouse Sialoglycoproteins	CMAH KO mouse Sialoglycoproteins	TRUE	OVA	100	IgG	1.0 (ug/mL)	NOT STATED	NOT STATED	NOT STATED	2.7

**Sæthre M, Baumann BC, Fung M, Seebach JD, Mollnes TE. Characterization of natural human anti-non-Gal antibodies and their effect on activation of porcine gal-deficient endothelial cells. TRANSPLANTATION. 2007;84(2):244-50.**	2007	Xenotransplantation	HD antigen as a potential target for 'non-gal' xenoreactive antibodies to porcine endothelial cells	19	Serum	Europe	ELISA	Synthetic	Neu5Gc-PAA	Neu5Ac-PAA	TRUE	BSA	100	IgG, IgM	70 (% of standard)	NOT STATED	90 (% of standard)	NOT STATED	7.714
**Saha S, Coady A, Sasmal A, Kawanishi K, Choudhury B, Yu H, et al. Exploring the Impact of Ketodeoxynonulosonic Acid in Host-Pathogen Interactions Using Uptake and Surface Display by Nontypeable Haemophilus influenzae. mBio. 2021;12(1).**	2021	Infection	Anti-Sia antibody responses mounted in humans following exposure to Sias on bacteria. Main focus on Ketodeoxynonulosonic acid, but also looked at Neu5Gc	24	Serum	North America	Glycan Array	Synthetic	Neu5Gc-containing glycans	NONE	FALSE	OVA	100	IgG, IgM	320 (RFU)	NOT STATED	50 (RFU)	NOT STATED	2.5
**Salama A, Evanno G, Lim N, Rousse J, Le Berre L, Nicot A, et al. Anti-Gal and Anti-Neu5Gc Responses in Nonimmunosuppressed Patients After Treatment With Rabbit Antithymocyte Polyclonal IgGs. Transplantation. 2017;101(10):2501-7.**	2017	Biotherapeutics	Anti-Neu5Gc antibodies in type 1 diabetes patients treated with rabbit anti-thymocyte globulin (ATG)	58	Serum, stored at -80C	North America	ELISA	Natural	WT Mouse Sialoglycoproteins	CMAH KO mouse Sialoglycoproteins	TRUE	OVA	100	IgG, IgM	3.5 (ug/mL)	NOT STATED	0.65 (ug/mL)	NOT STATED	285.7

**Samraj AN, Bertrand KA, Luben R, Khedri Z, Yu H, Nguyen D, et al. Polyclonal human antibodies against glycans bearing red meat-derived non-human sialic acid N-glycolylneuraminic acid are stable, reproducible, complex and vary between individuals: Total antibody levels are associated with colorectal cancer risk. PLoS One. 2018;13(6):e0197464.**	2018	Cancer	Links between anti-Neu5Gc antibody titres and colorectal cancer risk in NHS professionals. Also assessed links between red meat intake and anti-Neu5Gc antibodies.	1469	Serum, collected in heparin-treated or EDTA-treated tubes then centrifuged. Stored in liquid nitrogen	North America	ELISA	Natural	WT Mouse Sialoglycoproteins	CMAH KO mouse Sialoglycoproteins	TRUE	OVA	90	IgG	1.7 (ug/mL)	NOT STATED	NOT STATED	0.941	NOT STATED
**Scobie L, Padler-Karavani V, Le Bas-Bernardet S, Crossan C, Blaha J, Matouskova M, et al. Long-Term IgG Response to Porcine Neu5Gc Antigens without Transmission of PERV in Burn Patients Treated with Porcine Skin Xenografts. JOURnaL OF IMMUNOLOGY. 2013;191(6):2907-15.**	2013	Xenotransplantation	Anti-Neu5Gc antibodies in burn patients treated with porcine skin grafts	10	Serum	Europe	ELISA	Natural	WT Mouse Sialoglycoproteins	NONE	FALSE	OVA	60	IgG, IgM	8.0 (ug/mL)	NOT STATED	1.9 (ug/mL)	0.1875	NOT STATED

**Segatori VI, Cuello HA, Gulino CA, Albertó M, Venier C, Guthmann MD, et al. Antibody-dependent cell-mediated cytotoxicity induced by active immunotherapy based on racotumomab in non-small cell lung cancer patients. CANCER IMMUNOLOGY IMMUNOTHERAPY. 2018;67(8):1285-96.**	2018	Cancer	Anti-Neu5Gc-GM3 antibodies in serum of NSCLC patients immunized with Racotumomab (Anti-idiotype antibody which aims to trigger an immune response against Neu5Gc-GM3)	30	Serum	North America	ELISA	Natural	Neu5Gc-GM3	Neu5Ac-GM3	TRUE	HSA	56.6	IgG, IgM	130 (% of control)	NOT STATED	250 (% of control)	NOT STATED	5.19
**Senage T, Paul A, Le Tourneau T, Fellah-Hebia I, Vadori M, Bashir S, et al. The role of antibody responses against glycans in bioprosthetic heart valve calcification and deterioration. nat Med. 2022;28(2):283-94.**	2022	Xenotransplantation	Potential role of anti-Neu5Gc antibodies in calcification of bioprosthetic heart valves. Assessed titres pre- and post-transplantation	497	Serum	Europe	ELISA	Natural	WT Mouse Sialoglycoproteins	NONE	FALSE	OVA	100	IgG	2.9 (ug/mL)	NOT STATED	NOT STATED	0.069	NOT STATED

**Smart I, Goecke T, Ramm R, Petersen B, Lenz D, Haverich A, et al. Dot blots of solubilized extracellular matrix allow quantification of human antibodies bound to epitopes present in decellularized porcine pulmonary heart valves. Xenotransplantation. 2021;28(1):e12646.**	2021	Xenotransplantation	Comparison of dot blot and ELISA methods to measure human serum antibody binding to porcine heart valve tissues	12	Serum collected in Serum Monovettes and centrifuged for 2500xg for 20 min. stored at -80C	Europe	Dot blot	Natural	Solubilised GGTA1 KO porcine heart valves	Solubilised WT porcine heart valves	FALSE	NONE	16	Total	100 (AU)	NOT STATED	NOT STATED	0.514	NOT STATED
**Smart I, Goecke T, Ramm R, Petersen B, Lenz D, Haverich A, et al. Dot blots of solubilized extracellular matrix allow quantification of human antibodies bound to epitopes present in decellularized porcine pulmonary heart valves. Xenotransplantation. 2021;28(1):e12646.**	2021	Xenotransplantation	Comparison of dot blot and ELISA methods to measure human serum antibody binding to porcine heart valve tissues	12	Serum collected in Serum Monovettes and centrifuged for 2500xg for 20 min. stored at -80C	Europe	ELISA	Synthetic	Neu5Gc-PAA	Neu5Ac-PAA	TRUE	Polyethylene Glycol 600	66.6	Total	2.7 (Log titer)	NOT STATED	NOT STATED	NOT STATED	2.59

**Song KH, Kang YJ, Jin UH, Park YI, Kim SM, Seong HH, et al. Cloning and functional characterization of pig CMP-<i>N</i>-acetylneuraminic acid hydroxylase for the synthesis of <i>N</i>-glycolylneuraminic acid as the xenoantigenic determinant in pig-human xenotransplantation. BIOCHEMICAL JOURnaL. 2010;427:179-88.**	2010	Xenotransplantation	Human serum antibody binding to CMAH transfected pig kidney cells	NOT STATED	Serum	NOT STATED	Flow Cytometry	Natural	CMAH-transfected pig kidney cells	mock transfected pig kidney cells	TRUE	NONE	NOT STATED	IgG, IgM	10 (mean shift)	NOT STATED	30 (mean shift)	0.5	NOT STATED
**Tachi Y, Kobayashi T, Yokoyama I, Hayashi S, Negita M, namii Y, et al. Variability of Cytotoxicity to Pig Cultured Cells and Its Determinant Factor in Human Sera. Transplantation Proceedings. 1998;30(1):71-3.**	1998	Xenotransplantation	Assessing human serum cytotoxicity to PK15 pig kidney cell line as a potential indicator of graft rejection. Looked at HD antibody titres.	20	Serum, stored at -70C, not heat inactivated	Asia	ELISA	Natural	Neu5Gc-GM3	Uncoated wells	FALSE	OVA	10	IgG, IgM	0.09 (OD)	NOT STATED	0.072 (OD)	NOT STATED	0.189

**Tahara H, Ide K, Basnet NB, Tanaka Y, Matsuda H, Takematsu H, et al. Immunological property of antibodies against N-glycolylneuraminic acid epitopes in cytidine monophospho-N-acetylneuraminic acid hydroxylase-deficient mice. J Immunol. 2010;184(6):3269-75.**	2010	Healthy Population	Investigating anti-Neu5Gc antibodies in CMAH KO mice, but also assessed cytotoxicity of human serum antibodies against WT and CMAH KO mouse cells	7	Serum, heat inactivated	Asia	51 Cr release assay	Natural	51 Cr labelled WT mouse thymocytes	50 Cr labelled CMAH KO mouse thymocytes	TRUE	NOT STATED	NOT STATED	Total	10 (increase in % cytotoxicity)	NOT STATED	NOT STATED	0.5	NOT STATED
**Takiguchi M, Tamura T, Goto M, Kusakawa S, Milgrom F, Kano K. Immunological studies on Kawasaki disease. I. Appearance of Hanganutziu-Deicher antibodies. Clin Exp Immunol. 1984;56(2):345-52.**	1984	Inflammation	HD antibodies in serum from patients with Kawasaki disease	56	Serum, stored at -20C, heat inactivated	Asia	ELISA	Natural	High molecular weight bovine erythrocyte glycoprotein	NONE	FALSE	PBST	43% (IgM), 3% (IgG), 11% (IgA)	IgG, IgM, IgA	NOT STATED	NOT STATED	0.507 (OD)	NOT STATED	NOT STATED
**Tangvoranuntakul P, Gagneux P, Diaz S, Bardor M, Varki N, Varki A, Muchmore E. Human uptake and incorporation of an immunogenic nonhuman dietary sialic acid. Proc natl Acad Sci U S A. 2003;100(21):12045-50.**	2003	Healthy Population	Investigation of Neu5Gc incorporation into human tissues, including the presence of circulating anti-Neu5Gc antibodies	18	Serum	North America	ELISA	Synthetic	Neu5Gc-PAA	Neu5Ac-PAA	TRUE	TBST	88.9 (IgA), 77.8 (IgM), 94.4 (IgG)	IgG, IgM, IgA	0.26 (OD)	0.19 (OD)	0.12 (OD)	NOT STATED	3.077

**Taylor RE, Gregg CJ, Padler-Karavani V, Ghaderi D, Yu H, Huang S, et al. Novel mechanism for the generation of human xeno-autoantibodies against the nonhuman sialic acid N-glycolylneuraminic acid. J Exp Med. 2010;207(8):1637-46.**	2010	Healthy Population	Investigating induction of anti-Neu5Gc antibodies in humans, with a specific focus on presentation of Neu5Gc on NTHi bacteria.	15	Serum	South America	ELISA	Synthetic	Neu5Gca2-6GalB1-4GlcB-HSA	HSA only	FALSE	OVA	73 (IgM), 93 (IgG)	IgG, IgM	6.5 (ug/mL)	NOT STATED	4 (ug/mL)	NOT STATED	2.92
**Vanhove B, Duvaux O, Rousse J, Royer PJ, Evanno G, Ciron C, et al. High neutralizing potency of swine glyco-humanized polyclonal antibodies against SARS-CoV-2. European Journal of Immunology. 2021;51(6):1412-22.**	2021	Biotherapeutics	Potential issues with Neu5Gc-containing glycans on porcine antibodies used to neutralise SARS-CoV-2 infection	59	Serum	Europe	ELISA	Natural	GGTA1 KO pig IgG	CMAH KO pig IgG	FALSE	OVA	22	IgG	0.6 (OD decrease)	NOT STATED	NOT STATED	NOT STATED	2.3
**Wang ZY, Burlak C, Estrada JL, Li P, Tector MF, Tector AJ. Erythrocytes from GGTA1/CMAH knockout pigs: implications for xenotransfusion and testing in non-human primates. Xenotransplantation. 2014;21(4):376-84.**	2014	Xenotransplantation	Assessing human serum antibody response to Neu5Gc on porcine erythrocytes (GGTA1 KO and CMAH/GGTA1 KO)	11	Serum	North America	ELISA	Synthetic	Neu5Gc-PAA	Neu5Ac-PAA	TRUE	TBST	91	IgG, IgM	0.38 (ug/mL)	NOT STATED	0.085 (ug/mL)	NOT STATED	63.42

**Wang ZY, Li P, Butler JR, Blankenship RL, Downey SM, Montgomery JB, et al. Immunogenicity of renal microvascular endothelial cells from genetically modified pigs. Transplantation. 2016;100(3):533-7.**	2016	Xenotransplantation	Human serum antibody binding to GGTA1/CMAH KO porcine renal microvascular endothelial cells.	pooled	Serum	NOT STATED	Flow Cytometry	Natural	GGTA1 KO porcine RMECs	GGTA1/CMAH KO porcine RMECs	FALSE	BSA	NOT STATED	IgG, IgM	40000 (MFI change)	NOT STATED	10000 (MFI change)	NOT STATED	NOT STATED
**Yeh P, Ezzelarab M, Bovin N, Hara H, Long C, Tomiyama K, et al. Investigation of potential carbohydrate antigen targets for human and baboon antibodies. Xenotransplantation. 2010;17(3):197-206.**	2010	Xenotransplantation	Identifying potential non-gal xenoreactive epitopes on porcine cells, including Neu5Gc	16	Serum	North America	ELISA	Synthetic	Neu5Gc-PAA	PAA only	FALSE	BSA	NOT STATED	IgG, IgM	0.4 (OD)	NOT STATED	0.2 (OD)	1	NOT STATED
**Yoshino H, Miyatani N, Saito M, Ariga T, Lugaresi A, Latov N, et al. Isolated bovine spinal motoneurons have specific ganglioside antigens recognized by sera from patients with motor neuron disease and motor neuropathy. J Neurochem. 1992;59(5):1684-91.**	1992	Inflammation	Anti-Neu5Gc-GM1 antibodies in serum from patients with motorneuron disease	2	Serum	North America	Thin layer chromatography immunostaining	Natural	Bovine brain gangliosides	motorneuron gangliosides	FALSE	BSA	50	IgM	NOT STATED	NOT STATED	NOT STATED	NOT STATED	NOT STATED

**Zhang R, Wang Y, Chen L, Wang R, Li C, Li X, et al. Reducing immunoreactivity of porcine bioprosthetic heart valves by genetically-deleting three major glycan antigens, GGTA1/β4GalNT2/CMAH. Acta Biomater. 2018;72:196-205.**	2018	Xenotransplantation	Human serum antibody binding to GGTA1/CMAH/B4GalNT2 KO porcine heart valve tissue	Pooled	Commercial AB serum, heat inactivated	North America	Flow Cytometry	Natural	GGTA1 KO porcine PBMCs	GGTA1/CMAH KO porcine PBMCs	FALSE	Goat Serum	NOT STATED	IgG, IgM	3 (relative MFI decrease)	NOT STATED	10 (Relative MFI decrease)	0.33	NOT STATED
**Zhang Z, Hara H, Long C, Iwase H, Qi H, Macedo C, et al. Immune Responses of HLA Highly Sensitized and Nonsensitized Patients to Genetically Engineered Pig Cells. Transplantation. 2018;102(5):e195-e204**	2018	Xenotransplantation	Binding of serum antibodies from 'immune hypersensitive' renal failure patients to common pig xenoantigens as a predictor of graft failure	10	Serum, patients all received previous hemodialysis	North America	ELISA	Synthetic	Neu5Gc-PAA	PAA only	FALSE	BSA	25	IgG, IgM	0.08 (OD)	NOT STATED	0.01 (OD)	NOT STATED	0.25
**Zhu A, Hurst R. Anti-N-glycolylneuraminic acid antibodies identified in healthy human serum. Xenotransplantation. 2002;9(6):376-81.**	2002	Healthy Population	Assessing anti-Neu5Gc titres in healthy individuals as a source of 'non-Gal' xenoreactive antibodies	20	Serum	NOT STATED	Hemagglutination	Natural	porcine erythrocytes	porcine erythrocytes plus Neu5Gc	FALSE	NOT STATED	85	IgG	50 (average titer)	NOT STATED	NOT STATED	NOT STATED	0.8
